# Supplementary material for: Design of orthogonal genetic switches based on a crosstalk map of σs, anti-σs, and promoters
Source: Mol Syst Biol. 2013 Oct 29;9:702. doi: 10.1038/msb.2013.58 (PMC3817407; doi:10.1038/msb.2013.58)
Supplement: Supplementary Information — Supplementary Figures S1–S24 [file msb201358-s1.pdf]

Supplementary information for:

Design of orthogonal genetic switches based on a crosstalk map of σs, anti-σs, and promoters

Virgil A. Rhodius, Thomas H. Segall-Shapiro, Brian D. Sharon, Amar Ghodasara, Ekaterina Orlova, Hannah Tabakh, David H. Burkhardt, Kevin Clancy, Todd C. Peterson, Carol A. Gross, and Christopher A. Voigt

I. Promoter Identification and Design
- I.A. *Identification of ECF promoters using genomic information*
- I.B. *ECF σ promoter modeling*
- I.C. *Predicted orthogonality of the complete promoter, as well as the -10 and -35 regions*
- I.D. *σ70 promoter modeling and prediction*
- I.F. *Improving promoter response with synthetic UP elements*

II. σ and Anti-σ Library Characterization
- II.A. *Selecting ECF σs, anti- σs and promoters*
- II.B. *Complete σ screening data, including multiple σs from each subgroup and non-orthogonal data*
- II.C. *Full transfer functions and cytometry data for promoter induction.*
- II.D. *Complete anti-σ screening data*
- II.E. *Growth assays of σ and anti-σ expression*
- II.F. *Simultaneous expression of multiple σs*
- II.G. *Expression of a σ in Klebsiella oyctoca*
- II.H. *Characterization of induction systems*

III. Quantification of Anti-σ Threshold Control

IV. Creating Chimeric σs
- IV.A. *Design of chimeric σs and promoters*
- IV. B. *Chimeric σ characterization*

V. Plasmids

VI. Supplementary Tables

VII. References

I. Promoter Identification and Design

I.A. Identification of ECF promoters using genomic information

A three-step search strategy was used to maximize the identification of promoter sequences for each ECF σ subgroup and increase the number of promoters identified compared to the original work by Staron and co-workers. First, based on the observations that many ECF σ groups autoregulate their own gene expression[1] and that some σs may regulate their own anti-σ[2], promoter motifs were searched for in sequences directly upstream of the σ gene, σ operon, and cognate anti-σ gene. Second, upstream regulatory regions were extracted for all σs within each subgroup to maximize the ability to find over-represented motifs. Third, BioProspector[3] was used to identify over-represented motifs in these upstream regulatory regions. BioProspector is a 2-block motif search algorithm that is ideally suited for bacterial promoters with variable length spacers between the -10 and -35 motifs.

All ECF σs in subgroups 01-43 and their cognate anti-σs were identified from Staron and co-workers (their Table S5)[1]. To enable efficient retrieval of their upstream regulatory sequences, all 1232 complete bacterial genome sequences and annotations were downloaded from the NCBI FTP site (11/1/2010). Both σs and anti-σs were identified from these genomes based on the annotation supplied by Staron *et al*: source genome, gene ID (GI) and their listed amino acid sequence (σs only). From the 1736 listed ECF σs and cognate 1203 anti-σs listed by Staron *et al.*, 1329 σs and 880 anti-σs were successfully identified from the NCBI annotated genomes. The remaining σs and anti-σs were from genomes not listed in the NCBI database and therefore were not used in this analysis.

For each ECF σ subgroup, three libraries of upstream regulatory sequences were extracted from: 1) directly upstream of the σ gene; 2) directly upstream of the σ gene operon (σ operons were defined as all consecutive genes adjacent to the σ gene, in the same orientation and separated by less than 50 nt from each other); 3) directly upstream of the cognate anti-σ gene (if known). Most promoters occur near the start of genes but can be difficult to detect when searching long upstream regulatory sequences for over-represented motifs. To facilitate identification, different length upstream regulatory sequences were extracted for each library, from the start codon to 100, 150, 200 and 300 nt upstream. For each library, searches for over-represented motifs were performed using BioProspector with the short 100 nt upstream sequences first and then repeated with the successively longer sequences. Motif searches with BioProspector were performed only on the forward strand and the highest scoring motifs selected from 100 reinitializations. The search for 2-block motifs was typically of the form, *W7 w5 G18 g15*: where *W* and *w* denotes the length (nt) of the upstream and downstream blocks, respectively; and *G* and *g* denotes the maximum and minimum distances (nt) separating the two blocks, respectively. These parameters were varied iteratively to optimize the searches for different promoter motifs. From all the library, sequence and motif search combinations, the highest scoring 2-block motif was selected as the representative promoter motif for each ECF σ. These were typically from the 100 or 200 nt sequences upstream of the ECF σ gene or operon.

Promoters for ECF subgroups 05-10, 19, 27 and 32 listed in Staron *et al*. were not identified in our search. Subgroups 05-10 are not autoregulated[1] and the remaining subgroups only had a few σs with highly related upstream sequences, making it difficult to search for over-represented motifs. For all of these cases, the promoter sequences were obtained from Staron *et al*. and Bioprospector was used to redefine the -35 and -10 motifs. Promoter sequences and their -10/-35 motifs are listed in Supplementary Table S1.

I.B. ECF σ promoter modeling and prediction

For each ECF σ subgroup, the highest scoring 2-block motif identified by BioProspector was used to construct promoter models following the method described by Rhodius and Mutalik[4]. The upstream and downstream motif sequences were used to compile Position Weight Matrices (PWMs)[5] for the -35 and -10 motifs, respectively. Specifically, for the regions identified by BioProspector, the weights ($W_{b,i}$) for each position (i) and base (b) were computed as

$$W_{b,i}^{motif} = \ln\left[\frac{(n_{b,i}+0.5)/(N+2.0)}{P_b}\right] \quad , \qquad \text{(S1)}$$

where $n_{b,i}$ is the number of times that the base b is found at position i in the promoter set, N is the number of promoters in the promoter set, and P_b is the probability of finding a specific base at any given position (assumed to be 0.25). Bayesian pseudocounts of 0.5 were added to each base to represent the relative uncertainty in the promoter sequences. To evaluate a motif in a promoter, the appropriate weights can be summed for a given sequence of bases b at positions i to obtain a complete -35 or -10 score. Additionally, the variable distances between the -35 and -10 motifs were used to construct spacer length histograms and to calculate a penalty score for suboptimal spacer lengths,

$$S = \ln\left[\frac{f+0.005F}{F+0.005F}\right] \quad , \qquad \text{(S2)}$$

where F is the frequency of the most commonly observed (assumed to be optimal) spacer length in the promoter set, and f is the frequency of the spacer length in the promoter being evaluated. Bayesian pseudocounts of 0.5% of the frequency of the optimal spacer length were added to account for uncertainty.

The total promoter score was calculated as a sum of the -35 and -10 motifs evaluated with PWMs and the spacer length penalty

$$Score = \sum_{i=1}^{L_{-35}} W_{b,i}^{-35} + \ln\left[\frac{f+0.0005F}{F+0.0005F}\right] + \sum_{i=1}^{L_{-10}} W_{b,i}^{-10} \quad . \qquad \text{(S3)}$$

When visualizing motifs, the sequence logos of aligned promoter sequences were generated using WebLogo 3 (http://weblogo.threeplusone.com; composition set to 50% GC[6]) for Figure 2, and WebLogo 2.8.2 (http://weblogo.berkeley.edu/; no small sample correction) for Supplementary Figure S1. For the Weblogos to compensate for the variable spacing between the -35 and -10 motifs for each promoter model, the distances between them was fixed to the most commonly observed spacer length. Figure 2 focuses on the -35 and -10 regions. Figure S1 contains the complete information for the promoter models, including more of the sequence flanking the -35 and -10 motifs, as well showing the distance from the promoter to the downstream target gene.

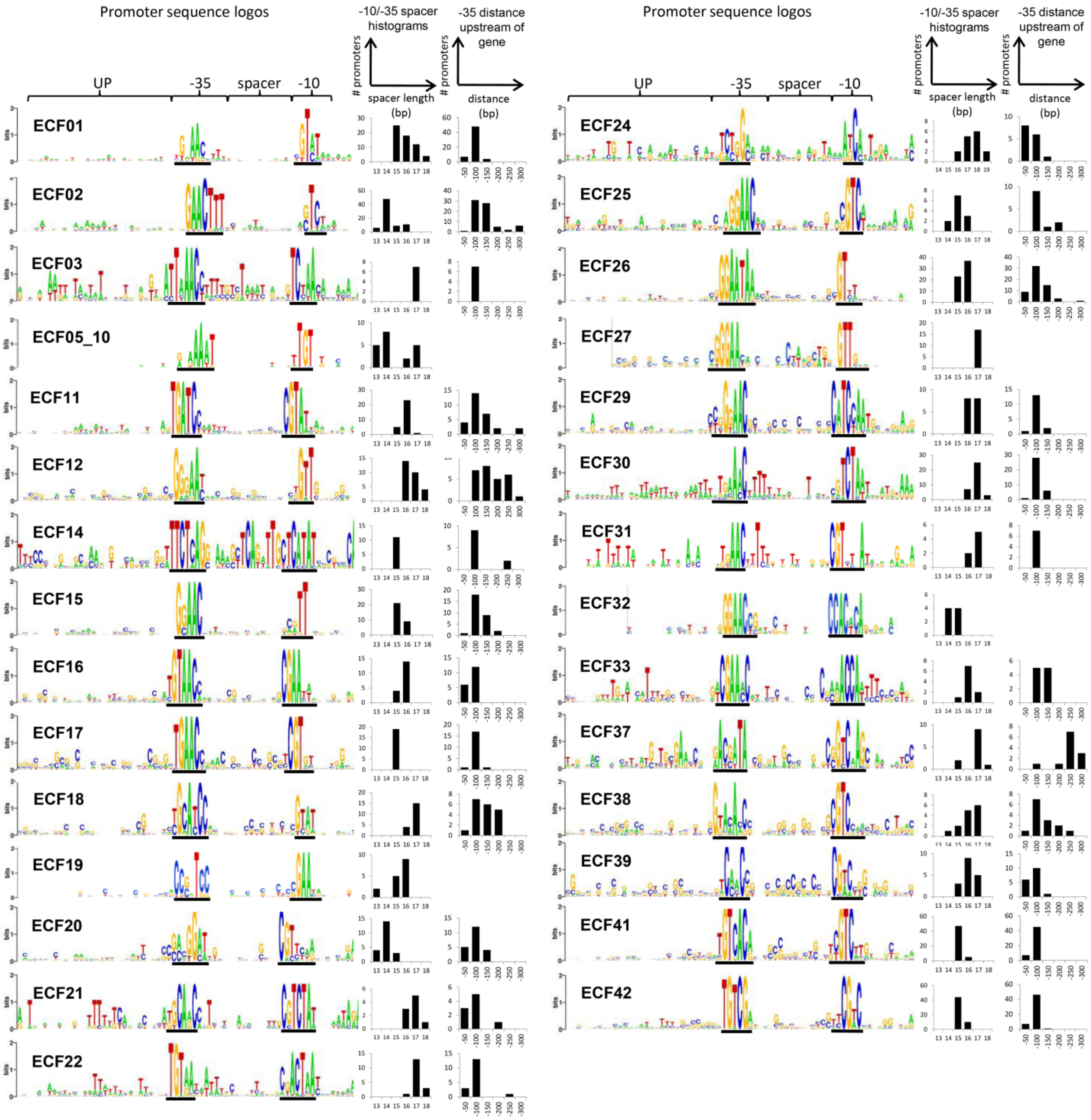

Figure S1: ***Complete promoter models are shown for each ECF subgroup.*** *The models contain a sequence logo illustrating the upstream (UP) sequence, -35 sequence, spacer sequence, -10 sequence, and 10 bases following the -10. The histograms show, from all the analyzed promoters, the distance between the -35 and -10 motifs, and the distance between the -35 motif and the nearest downstream gene. The exact -35 and -10 sequences identified by the 2-block search algorithm, BioProspector, are underlined underneath each sequence logo, and were used to calculate the distances for the -10/-35 spacer histograms.*

I.C. Predicted orthogonality of the promoters in the library, as well as of their individual -35 and -10 regions.

The 29 generated promoter models were used to analyze all 706 promoters in the promoter library. This analysis revealed a high level of predicted orthogonality between the ECF subgroups. A similar analysis was performed on just the -10 or -35 subsites, revealing far less predicted orthogonality. Equation S3 was used to evaluate the full promoters, and the first or third term of that equation was used for the -35 of -10 subsite analysis as appropriate.

Z-scores were calculated from the full, -35, and -10 promoter scores (Figure S2) by normalizing to the predicted on-target promoter scores. The means and standard deviations of the scores for on-target promoters for each model were calculated, and the z-score calculated with the following equation

$$'Z - score' = \frac{<On-target\ scores> - Score}{\sigma_{On-target\ scores}} . \quad \text{(S4)}$$

Hence, the z-score for a given promoter represents the number standard deviations a promoter is from the mean score of the on-target promoter set.

The orthogonally of the full promoter model was quantitatively compared to the -10 and -35 models using Z-scores. First, the Z-scores were capped at a minimum of -3, as a promoter that is 3 or more standard deviations away from the on-target mean value has little chance to be active. Therefore, a score of less than -3 is should generally be equivalent to -3 when considering crosstalk. Next, for each promoter model, the mean of the Z-scores of all the off-target promoters in the promoter library was calculated. This was repeated using just the -35 and -10 models to get three sets of 29 mean off-target Z-scores.

While the mean values of the mean off-target scores are similar (full model= -2.91, -10 model= -2.48, -35 model= -2.72), the full model is shown to be significantly better at generating orthogonality. Two-tailed, paired t-tests between the set of mean off-target scores from the full model and the set of scores from the -10 or -35 models yield p values $<<0.05$ in both cases. This difference remains significant ($p<0.05$) for both the -10 and -35 model when not comparing pairwise by promoter model (ie, using an unpaired t-test), but it is not as strong for the -35 model. In all analyses, the -10 model is much worse at producing orthogonally than either the -35 model or the full model.

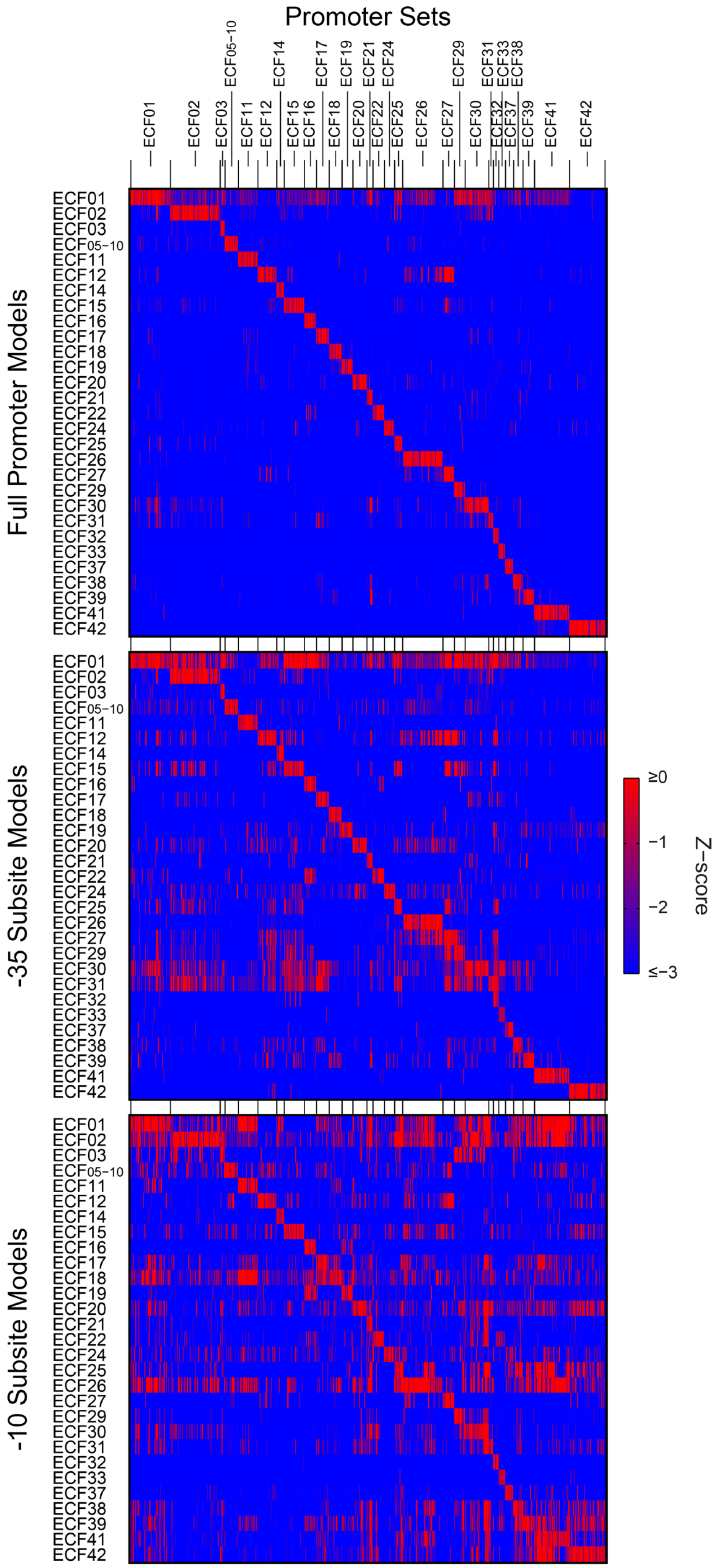

Figure S2: ***The predicted orthogonality of ECF σ promoter models and individual subsite models is shown.*** *Heatmap of Z-score analysis using the 29 promoter models to score all 706 identified ECF σ promoters. The blue to red color range covers Z-scores from -3 to 0, representing scores ranging from the mean on-target score to three standard deviations below. Z-score analysis demonstrates how the -35 and -10 promoter models are both needed for specificity.*

I.D. σ 70 promoter modeling and prediction

We built a σ 70 promoter model to screen promoter constructs for potential overlapping σ 70 promoter sequences. This model was used to determine whether the identified ECF σ promoters had a possible overlapping σ 70 binding site and therefore should not be included in our library (See II.A.). The σ 70 promoter model was constructed from 674 known σ 70 promoter sequences with experimentally determined transcription starts obtained from RegulonDB[7] (http://regulondb.ccg.unam.mx/). Since the -10 and -35 motifs of σ 70 promoters are poorly conserved, work by Shultzaberger *et al.* [8] was used as a guide for identifying the motifs. A 2-step search using the 1 block function of BioProspector was used. First, the -10 motif was identified as a 6 mer between positions -16 to -5 (a large window was used to allow for inaccuracies mapping the start site). Next, the -35 motif was identified as a 6 mer 15-20 nt upstream of the identified -10 motif. Four PWMs were constructed using the method of Rhodius and Mutalik[4]. As in Section I.C., a PWM_{-35} is built for the -35 motif (aTTGaca) and a PWM_{-10} for the -10 motif (TAtaaT). In addition, a PWM_{spacer} was built for a 10-mer block aligned from -21 to -13 aligned with the -10. This incorporates the putative Zn finger contact from the β' subunit of RNA polymerase (-21 to -18)[9], -17/-16 dyad and -15/-14 TG motif[10,11]. Finally, PWM_{start} is included to capture the transcription start site (-1/+1). All of these PWMs were built using Equation S1. Two spacer penalties were constructed[4] using Equation S2 based on distance histograms between the -35, -10 and start motifs: a spacer penalty (-35 to -10) and a discriminator penalty (-10 to +1). Upstream sequences were scored using counts of overlapping A- and T-tracts between positions -57 to -37, assuming the 5' end of the -35 motif is at position -36[12]. From these terms, the total σ 70 promoter score was calculated as:

$$Score = (UP\ model) + PWM_{-35} + PWM_{spacer} + PWM_{-10} + PWM_{start} + (Spacer\ penalty) + (Discriminator\ penalty) \tag{S5}$$

Inserting the terms described in Section I.B yields,

$$Score = (N_{AAA} + N_{TTT}) + \sum_{i=1}^{7} W_{b,i}^{-35} + \sum_{i=1}^{10} W_{b,i}^{spacer} + \sum_{i=1}^{6} W_{b,i}^{-10} + + \sum_{i=1}^{2} W_{b,i}^{start} + \ln\left[\frac{(f_{spacer} + 0.005F_{spacer})}{(F_{spacer} + 0.005F_{spacer})}\right] + + \ln\left[\frac{(f_{discrim} + 0.005F_{discrim})}{(F_{discrim} + 0.005F_{discrim})}\right] , \tag{S6}$$

where N_{AAA} is the number of AAAs, and N_{TTT} is the number of Ts proceeding the -35 site.

Note that this promoter model is more complex than that used for the ECF σs for several reasons. First, the additional PWM_{spacer} term is based on several contacts between σ^{70}-RNA polymerase and the promoter region that are not known to occur with ECF σs[9–11]. Second, the discriminator penalty and PWM_{start} scores rely on the correct identification of the transcriptional start site for each promoter. This was experimentally established for the σ70 promoters, but is unknown for the ECF σ promoters. Third, the UP model was not applied to the ECF σ promoter models for determining promoter orthogonality. This is because the upstream sequence does not distinguish promoter specificity between different ECF σs. However, the UP model was used to optimize ECF σ promoters for function in *E.* coli (see next section).

I.E. Improving promoters with synthetic UP elements

Promoter sequences were initially tested for activity against both cognate σs from their own ECF σ group. (See section II.A. for information on how these specific promoters and σs were selected.) Many non-functional promoter constructs were from GC-rich organisms and consequently had poor upstream sequences with little or no AAA- and TTT-tracts. These were scored by counting the number of overlapping AAA- and TTT-tracts within the sequence window -35 to -57 (assuming that the 5' end of the -10 motif is at position -10). For these promoters, the sequence between -60 to -35 was replaced with a synthetic UP-element derived from the upstream region of the Pecf02_2817 promoter; CATGACAAAATTTTTTAGATGCGTT, which generates a score of 6. The A- and T-tracts were designed predominantly in the proximal α binding site (-47 to -57) to mimic the location of the observed A- and T-rich sequences of the active σ promoters (data not shown). Adding the UP-element greatly increased the function of a number of the nonfunctional promoters (Figure S3), and the UP-element was added to all promoters except for those that proved functional without it in this test (Pecf02_2817, Pecf11_3726, Pecf16_3622, Pecf20_992, Pecf30_2079, Pecf31_34, Pecf32_1122, Pecf33_375). UP-element modified promoters were used in all following experiments.

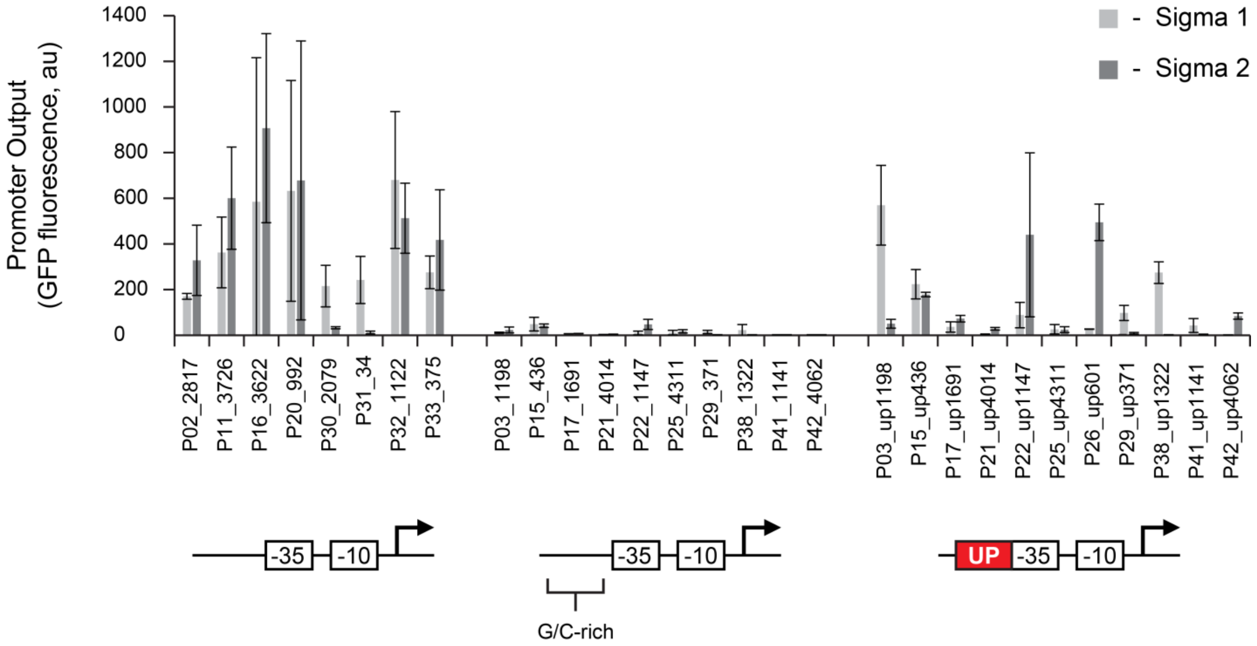

Figure S3: ***Improvement of promoter activity by adding UP-elements.*** *Promoter sequences were tested for activity against both cognate σs from their ECF σ group. (σ 1 denotes the σ with a lower number in the library, while σ 2 denotes the higher one. For example, with P02_2817, σ 1 is ECF02_915 and σ 2 is ECF02_2817. Inactive promoters tended to contain G/C-rich upstream sequences. These sequences were replaced with synthetic UP-element (CATGACAAAATTTTTTAGATGCGTT; -60 to -35), improving promoter activity. In vivo assays were performed by inducing σ expression with 100 μM IPTG for 6 hr and measuring promoter activity from GFP fluorescence using flow cytometry. Each bar represents the average promoter output from two independent assays, except for: P15_436 σs 1 and 2, P21_4014 σ 2, P25_4311 σs 1 and 2, P41_1141 σs 1 and 2, and P41_4062 σs 1 and 2, which are the average promoter output from three independent assays, and P21_4014 σ 1, P29_371 σs 1 and 2, and P38_1322 σs 1 and 2, which are the average promoter output from four independent assays. Error bars represent standard deviations.*

II. σ and Anti-σ Library Characterization

II.A. Selecting σs, anti-σs and promoters

Libraries of σs and anti-σs were constructed using several criteria. To maximize phylogenetic diversity, 2 σs were selected from each of the 43 ECF subgroups defined by Staron *et al*[1]. to create a library of 86 σs. Within each subgroup, σs were preferentially selected from genomes closely related to *E. coli* to maximize the likelihood of binding to *E. coli* RNAP. Since some ECF subgroups only contain σs from genomes phylogenetically distant to *E. coli* this still resulted in a σ library spanning 6 bacterial classes. σs were also selected if they had a known cognate anti-σ[1]. Not all σs have known anti-σs; however, this enabled the creation of a library of 62 anti-σs cognate to 62 σs from the main σ library.

Where possible, a given σ was paired with the putative promoter for that σ from the same genome. In these cases the promoter and σ have the same unique ID (*e.g.*, ECF02_2817 and $P_{02\text{-}2817}$). However, this pairing was not always possible and several criteria were used in selecting the final promoters for each σ group:

1) Promoters were preferentially selected that were discovered upstream of σ genes also present in our σ library.
2) Preference was given to promoters that were predicted to be orthogonal against the other ECF σs: *i.e.* scored highly in their own promoter model and scored poorly against the other promoter models.
3) Promoters were also screened against any overlapping host promoter sequences using an *E. coli*-specific σ^{70} promoter model for the housekeeping σ and the ECF05-10 promoter model for FecI. This was especially important for promoters selected from A/T-rich genomes, since they often contained weak overlapping σ^{70} promoter signals that are also A/T-rich.

All promoters were named using the convention $P_{\mathbf{XX\_YYYY}}$, where "XX" and "YYYY" denotes the subgroup and unique ID of the downstream parent σ gene (*e.g.,* P_{02_2817} is the promoter upstream of σ ECF02_2817).

II.B. Complete σ screening data, including multiple σs from each subgroup and non-orthogonal data

After promoter optimization, activity assays were performed combinatorially between all optimized promoters and all members of the σ library (Figure S4). For each promoter, cells containing the promoter-gfp construct were transformed with the entire σ library in 96 well format, recovered, induced, and fluorescence measured with flow cytometry. Fluorescence measurements were compared to controls lacking σs (but including the promoter-gfp construct) to calculate fold induction (Methods). This testing was used to identify the active σs and promoters in the library, even in cases where the promoter models did not match their intended subgroup. Additionally, these results allowed the selection of a subset of orthogonal σ:promoter pairs, which could be used in the same engineered system without crosstalk. The orthogonal subset of this data is shown in Figure 3e.

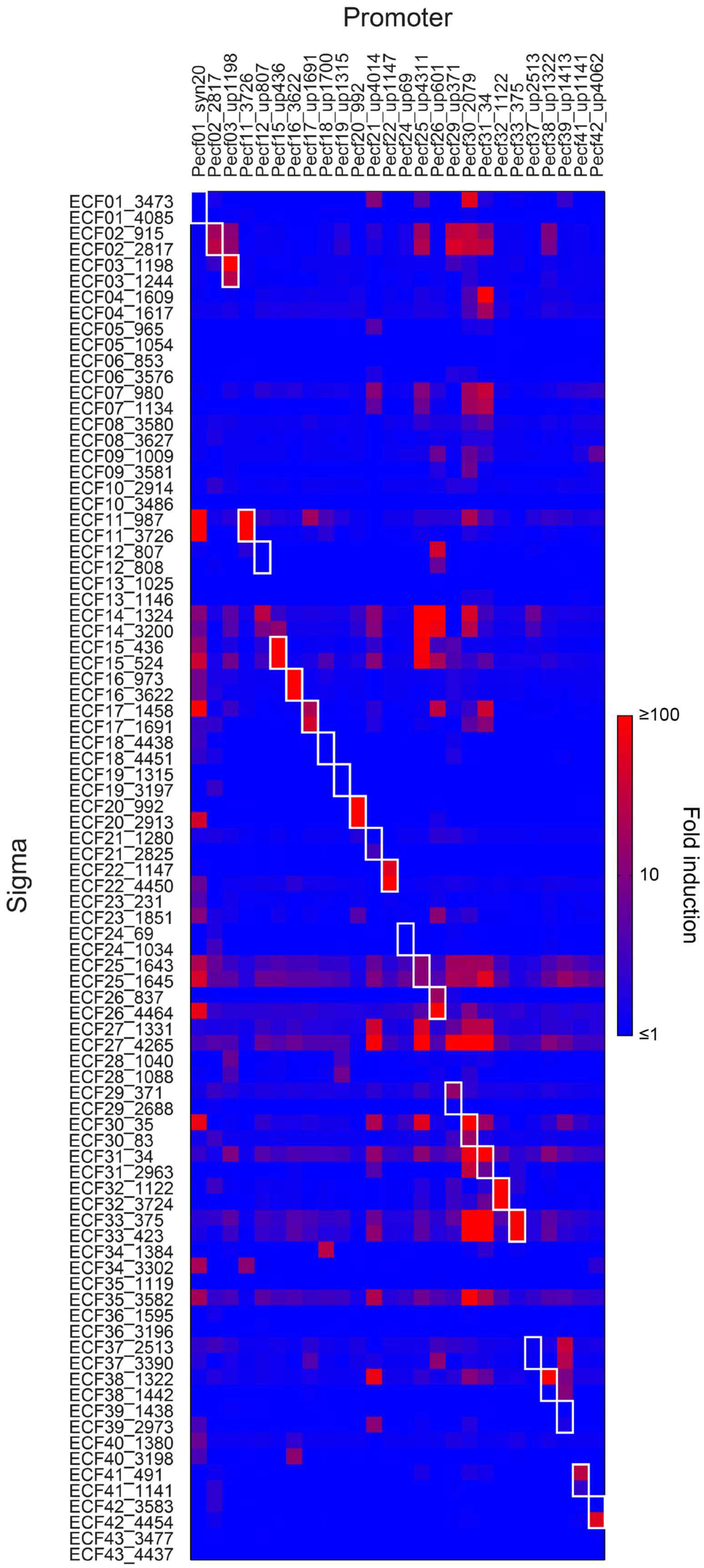

***<u>Figure S4:</u>* Activity of all σs with all active promoters.** *A heatmap of in vivo activities for the complete library of 86 σs tested against 26 active promoters is shown. Assays were performed by inducing σ expression with 100 μM IPTG for 6 hr and measuring promoter activity from GFP fluorescence using flow cytometry. White boxes indicate predicted cognate σ:promoter pairs. Each square represents the average fold induction from at least two independent assays.*

II.C. Full transfer functions and cytometry data for promoter induction.

Based on the combinatorial σ:promoter matrix (Figure S4), 58 members of the σ library were found to activate a promoter by at least 5-fold. Of these, 52 were chosen for further testing. Each of these 52 σs was paired with its most active promoter from the combinatorial assay, and induced at multiple levels of IPTG to determine promoter activity at multiple levels of σ (*i.e.*, the induction curve) (Figure S5). Measurements were performed in a similar manner to the combinatorial assay, at 0, 10, 20, and 50 µM IPTG in addition to 100 µM. These induction curves show a wide range of activities and are generally as expected. A subset of this induction curve data consisting of one member from each active ECF subgroup is shown in Figure 3b.

Figure S5 (next page): **σ titration assays.** *Each of the chosen 52 active σs was induced at 0, 10, 20, 50 and 100 μM IPTG with their most active cognate promoter in vivo. Assays were performed with 6 hr induction and promoter activity measured from GFP fluorescence using flow cytometry. Plots represent the average promoter activity from three independent assays and error bars represent one standard deviation. Red hash marks indicate toxicity (as judged by a fall in the 8hr OD600 to 75% or lower of wild type; see Section II.D. and Fig. S8; Note that growth measurements were only taken at 0, 10, 20, and 100 μM IPTG) occurring at different levels of IPTG induction. Cytometry distributions show typical log-scale histograms at 0 (black) and 100 μM IPTG (green). One σ:promoter pair, ECF03_1244:Pecf03_up1198, showed extreme toxicity at full induction, so three histograms are shown: black = 0 μM, green = 20 μM, red = 100 μM. All cytometry histograms are shown with log scaling and normalized to the mode of the distribution. A portion of the cells falls on the Y-axis and is not visible when plotted in this way. Light blue labels indicate that that σ:promoter set was included in the orthogonal subset of the σ library (Figure 3e).*

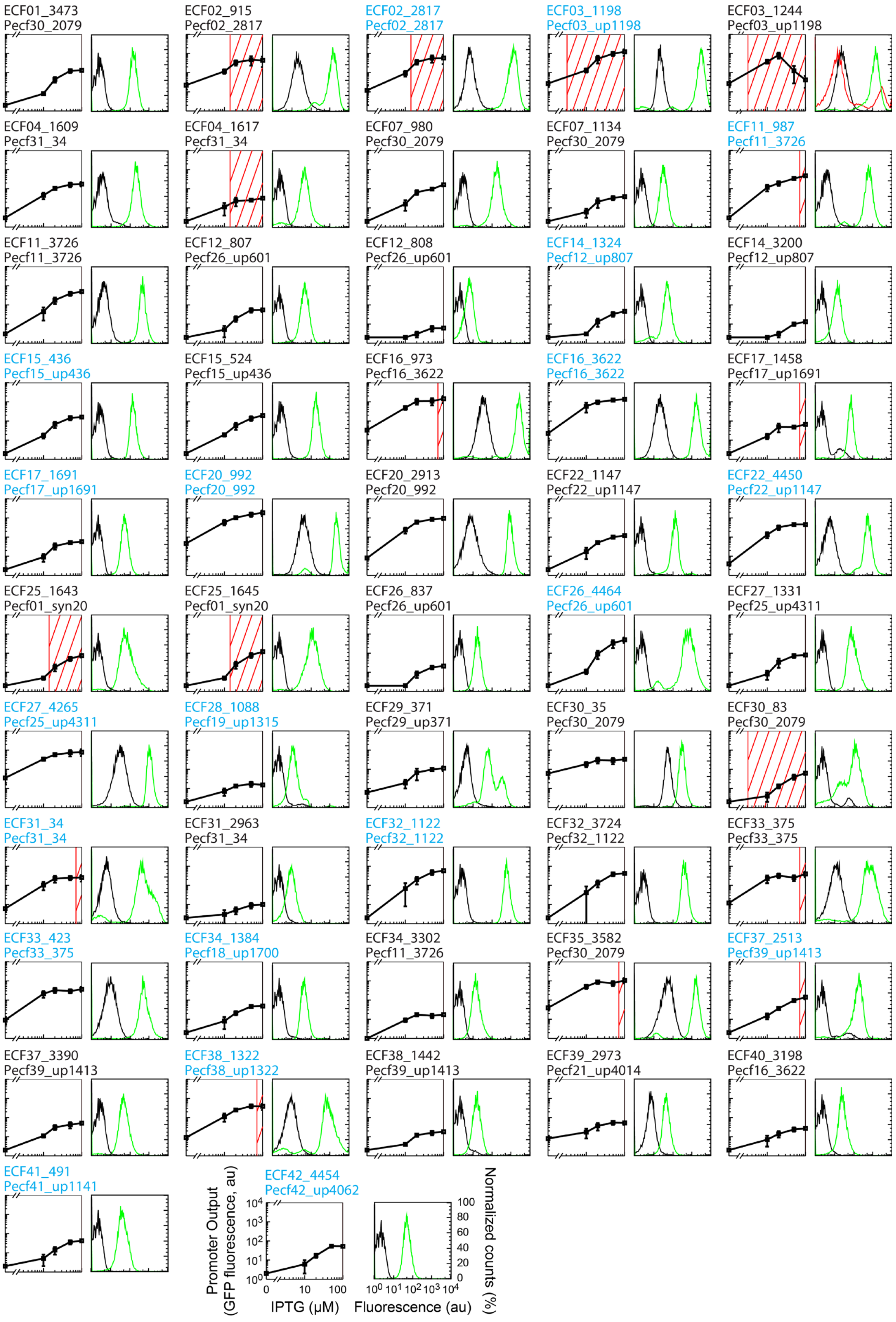

ECF01_3473
Pecf30_2079
ECF02_915
Pecf02_2817
ECF02_2817
Pecf02_2817
ECF03_1198
Pecf03_up1198
ECF03_1244
Pecf03_up1198
ECF04_1609
Pecf31_34
ECF04_1617
Pecf31_34
ECF07_980
Pecf30_2079
ECF07_1134
Pecf30_2079
ECF11_987
Pecf11_3726
ECF11_3726
Pecf11_3726
ECF12_807
Pecf26_up601
ECF12_808
Pecf26_up601
ECF14_1324
Pecf12_up807
ECF14_3200
Pecf12_up807
ECF15_436
Pecf15_up436
ECF15_524
Pecf15_up436
ECF16_973
Pecf16_3622
ECF16_3622
Pecf16_3622
ECF17_1458
Pecf17_up1691
ECF17_1691
Pecf17_up1691
ECF20_992
Pecf20_992
ECF20_2913
Pecf20_992
ECF22_1147
Pecf22_up1147
ECF22_4450
Pecf22_up1147
ECF25_1643
Pecf01_syn20
ECF25_1645
Pecf01_syn20
ECF26_837
Pecf26_up601
ECF26_4464
Pecf26_up601
ECF27_1331
Pecf25_up4311
ECF27_4265
Pecf25_up4311
ECF28_1088
Pecf19_up1315
ECF29_371
Pecf29_up371
ECF30_35
Pecf30_2079
ECF30_83
Pecf30_2079
ECF31_34
Pecf31_34
ECF31_2963
Pecf31_34
ECF32_1122
Pecf32_1122
ECF32_3724
Pecf32_1122
ECF33_375
Pecf33_375
ECF33_423
Pecf33_375
ECF34_1384
Pecf18_up1700
ECF34_3302
Pecf11_3726
ECF35_3582
Pecf30_2079
ECF37_2513
Pecf39_up1413
ECF37_3390
Pecf39_up1413
ECF38_1322
Pecf38_up1322
ECF38_1442
Pecf39_up1413
ECF39_2973
Pecf21_up4014
ECF40_3198
Pecf16_3622
ECF41_491
Pecf41_up1141
ECF42_4454
Pecf42_up4062
Promoter Output
(GFP fluorescence, au)
10^4
10^3
10^2
10^1
10^0
0
10
100
IPTG (μM)
10^0 10^1 10^2 10^3 10^4
Fluorescence (au)
100
80
60
40
20
0
Normalized counts (%)

II.D. Complete anti-σ screening data

This section outlines the initial screen for anti-σ activity; more detailed titration curves for those deemed active are presented in Section III. Of the 58 σs shown to activate a promoter by more than 5-fold (Figure S5), 47 have cognate anti-σs in the synthesized library. Based on the strength and orthogonality of the σ:promoter interaction, the most promising 35 anti-σs were chosen for further testing. To check for anti-σ activity, titrations of the σ and anti-σ were performed with the promoter:reporter construct most activated by the σ. These assays were performed using four levels of induction for the σ (0, 5, 20, and 100 μM IPTG), and three for the anti-σ (0, 10, and 50 nM HSL) in addition to a control lacking the anti-σ expression plasmid. This test showed that 32 of the 35 tested anti-σ were able to repress their cognate σ by at least 2-fold (Figure S6).

The 25 anti-σs with the best repression of their cognate σ from the titration assay were chosen for combinatorial orthogonality testing (Figure S7). In this test, the set of 25 anti-σs plus a no anti-σ control was tested against the 25 σ:promoter pairs targeted by the anti-σs. In order to better observe any repression effects, the σs were induced to an intermediate level (10 μM IPTG), while the anti-σs were induced to a high level (50 nM HSL). This assay shows that a number of the anti-σ:σ interactions appear to be fairly orthogonal. However, there are also a number that were less specific and affected many σs. These less specific anti-σs often greatly reduce growth (See Section II.D.).

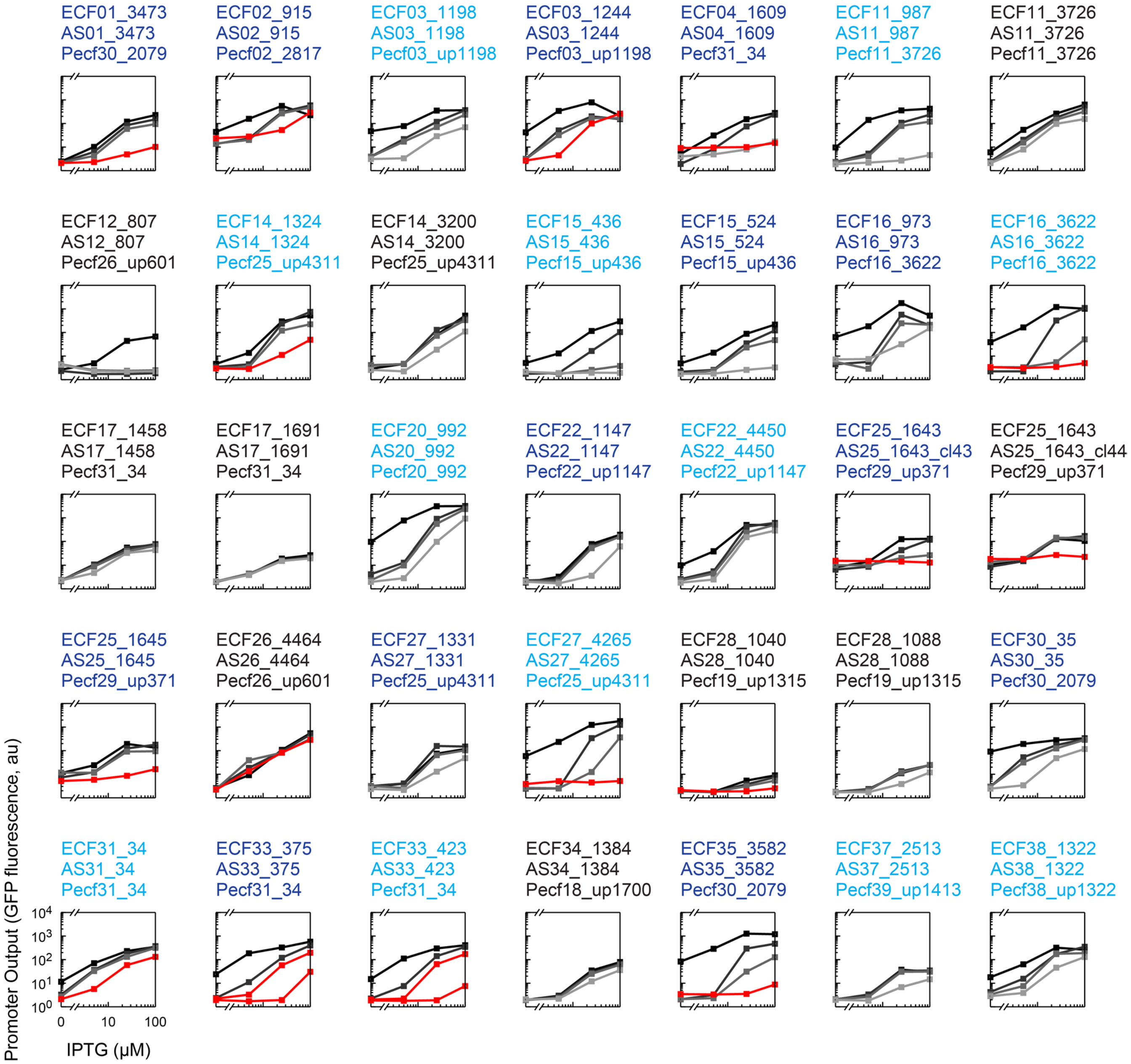

Figure S6: ***Anti-σ titration assays.*** *Transfer functions are shown for 35 anti-σ:σ:promoter sets. Promoter activity was measured across a range of anti-σ and σ inductions. For each assay: inducing the anti-σ; no anti-σ control, anti-σ with 0, 10 and 50 nM HSL(black, dark gray, mid-gray and light gray lines, respectively); from left to right inducing σ; 0, 5, 20 and 100 μM IPTG. Red lines indicate that level of anti-σ induction was toxic (as judged by a fall in the 8hr OD600 to 75% or lower of wild type; see Section II.D. and Fig. S9). Assays were performed in vivo with a 6 hour induction and promoter activity determined by measuring the fluorescence of sfgfp with flow cytometry. Data is from a single replicate. Light blue titles indicate that the anti-σ σ:promoter set was active and targets one of the σs in the orthogonal subset (these are included in Figure 5c). Dark blue titles indicate that the anti-σ was one of the 25 tested in the combinatorial assay (Figure S7).*

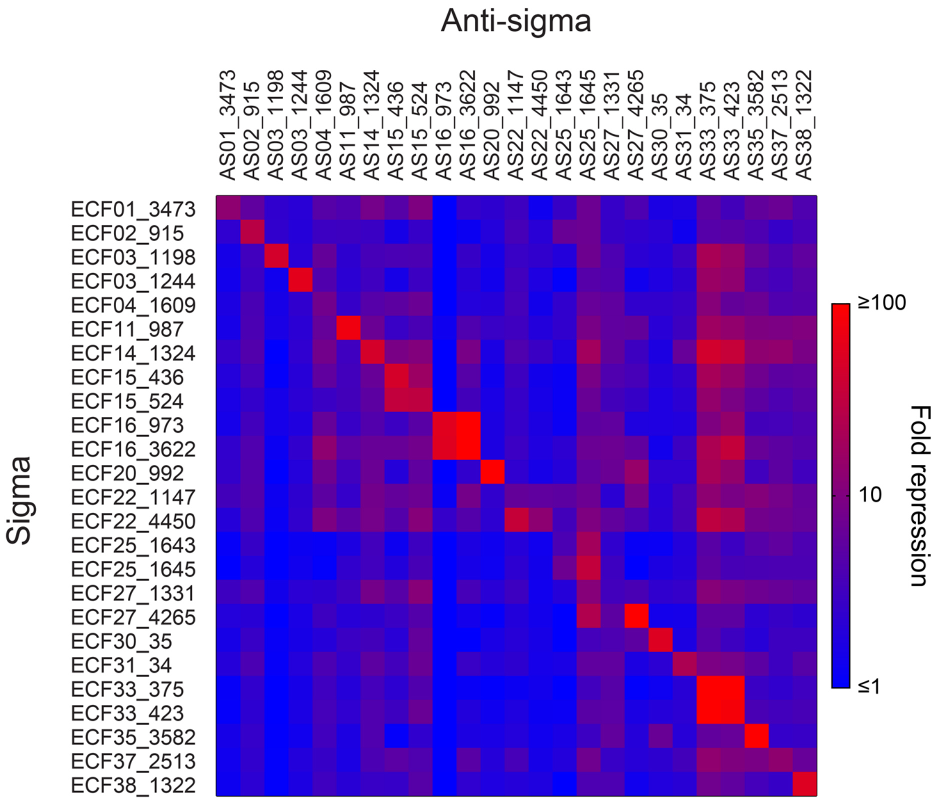

<u>Figure S7</u>: ***Repression of σs by all tested anti-σs.*** *A heatmap of fold repression of σ activity by different anti-σs is shown. Assays were performed by inducing σ expression with 10 μM IPTG and anti-σ expression with 50 nM HSL for 6 hr and fold repression calculated from fluorescence measurements. Each square represents the average fold repression from at least two independent assays.*

II.E. *σ and anti-σ library growth assays*

Both the σ and anti-σ libraries were tested for toxic effects occurring with expression in *E. coli* DH10β. Toxicity can be due to aberrant gene expression or titration of host RNAP by the σs, or by interaction of the anti-σs with essential host σs such as σ^E. The effects of expressing the σs and anti-σs were measured using 3 types of growth assays across a range of inductions: 1) transition phase culture density in liquid LB media; 2) exponential growth rates in liquid LB media; 3) colony size on LB agar plates (Figures 3c; S8; S9; Supplemental Tables S2.4, S2.5). For each condition, growth assays were performed from at least 3 separate transformations and across a range of inducer concentrations: 0, 10, 20 and 100 μM IPTG for the σ library; 0, 10 and 50 nM HSL for the anti-σ library. The σ library assay strains were freshly transformed *E. coli* DH10β cells carrying pN565 with the pVRa plasmid library and plasmid pET21a (Novagen) as a no σ control; the anti-σ library assay strains were *E. coli* DH10β cells freshly transformed with the pVRc plasmid library and pACYC184[13] as a no anti-σ control.

Under low levels of induction (10 μM IPTG or 10 nM HSL for the σ and anti-σ libraries, respectively) 95% of the σ library and 86% of the anti-σ library exhibited near wild type growth levels by all metrics (>75% wild type growth). Under high induction levels (100 μM IPTG or 50 nM HSL for the σ and anti-σ libraries, respectively) most growth defects were observed during transition phase and by colony size. For the σ library, 99% exhibited near wild type growth levels (>75% wild type growth) during exponential growth, whilst 77% and 90% exhibited near wild type growth measured in transition phase or by colony size, respectively. A similar pattern was observed with the anti-σ library but with slightly larger defects: 83%, 49% and 46% exhibited near wild type growth levels during exponential growth, in transition phase and by colony size, respectively. In general, transition phase and colony size yield a similar pattern of growth defects in both states across the σ and anti-σ libraries, likely due to the transition/stationary phase growth properties of cells in the center of colonies.

Both σs from subgroup 02 exhibited the highest toxicity. *E. coli* σ^E is also from subgroup 02 and is represented by the candidate ECF02_2817 in the σ library. *E. coli* σ^E is toxic when highly expressed[14]; consequently, the toxic effects of high expression of both ECF02 σ members in the library (ECF02_2817 and ECF02_915) suggest similar function. *E. coli* σ^E is also essential[15,16]; accordingly, high expression of its cognate anti-σ AS02_2817 is lethal due to repression of host σ^E activity. Interestingly, high expression of anti-σ AS02_915 from the same subgroup only gave reduced growth levels, suggesting that this anti-σ has reduced specificity for host σ^E. Both σ pairs from subgroups 03 and 25, and anti-σ pairs from subgroups 19, 33 and 35 were also highly toxic (<50% wild type growth), indicating similar activities of each member within the subgroup. There were also several instances of where just one subgroup member was toxic, indicating different functionality in an *E. coli* host (*e.g.* ability to bind *E. coli* RNAP). Importantly, the lack of toxicity of most library members suggests that they could have utility as orthogonal regulators in *E. coli*.

Figure S8 (next page): ***σ transition phase growth assay.*** *OD_{600} measurements were taken after 8 hours of growth at different levels of expression of each of the σs in the library. Plots represent the average measurement from three independent assays normalized to cells not expressing a σ, and error bars represent one standard deviation. Dark blue titles indicate 'active' σs with more than 5-fold induction of a promoter in the library, and light blue titles indicate a σ in the final orthogonal set (Figure 3e).*

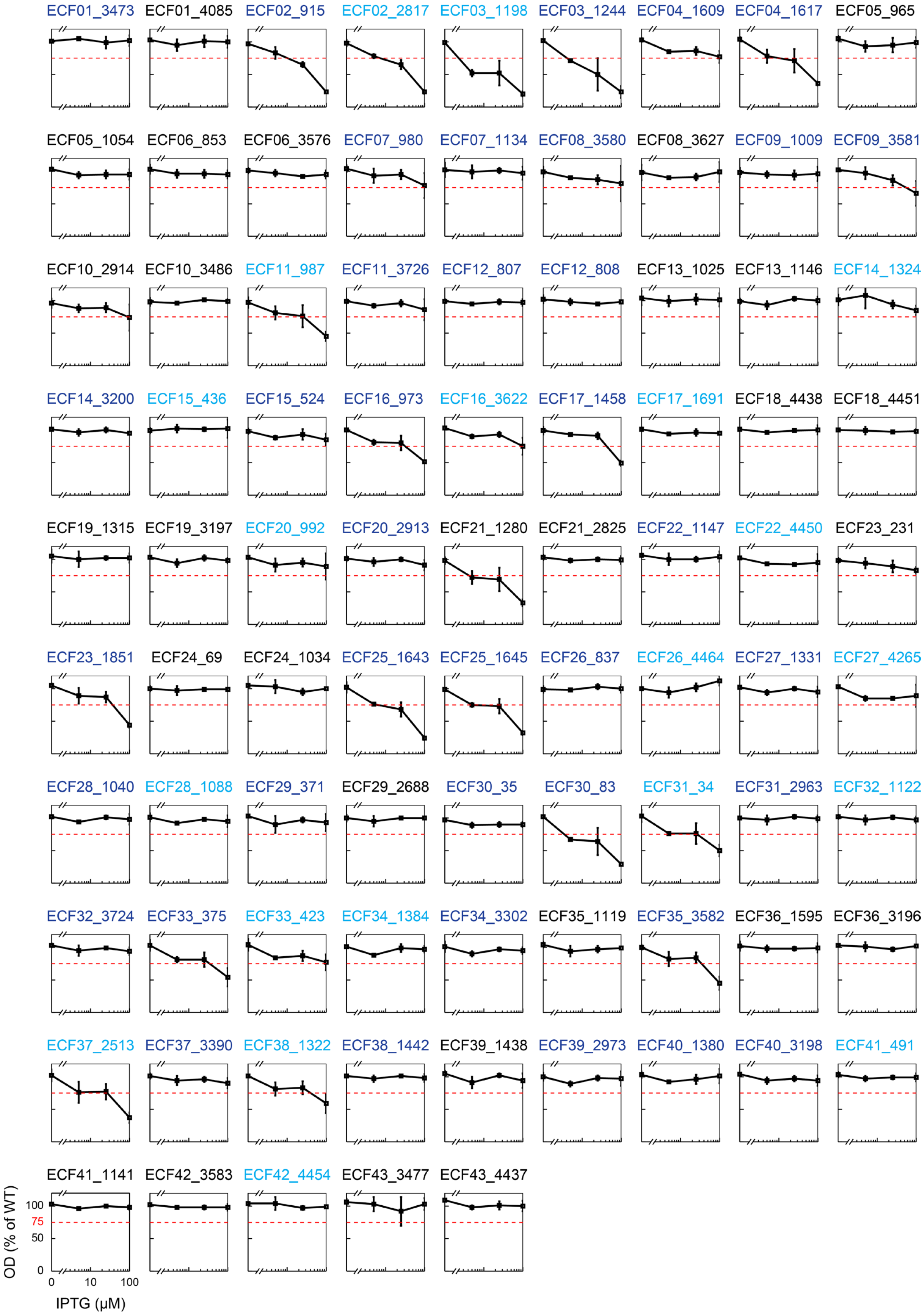
ECF01_3473
ECF01_4085
ECF02_915
ECF02_2817
ECF03_1198
ECF03_1244
ECF04_1609
ECF04_1617
ECF05_965
ECF05_1054
ECF06_853
ECF06_3576
ECF07_980
ECF07_1134
ECF08_3580
ECF08_3627
ECF09_1009
ECF09_3581
ECF10_2914
ECF10_3486
ECF11_987
ECF11_3726
ECF12_807
ECF12_808
ECF13_1025
ECF13_1146
ECF14_1324
ECF14_3200
ECF15_436
ECF15_524
ECF16_973
ECF16_3622
ECF17_1458
ECF17_1691
ECF18_4438
ECF18_4451
ECF19_1315
ECF19_3197
ECF20_992
ECF20_2913
ECF21_1280
ECF21_2825
ECF22_1147
ECF22_4450
ECF23_231
ECF23_1851
ECF24_69
ECF24_1034
ECF25_1643
ECF25_1645
ECF26_837
ECF26_4464
ECF27_1331
ECF27_4265
ECF28_1040
ECF28_1088
ECF29_371
ECF29_2688
ECF30_35
ECF30_83
ECF31_34
ECF31_2963
ECF32_1122
ECF32_3724
ECF33_375
ECF33_423
ECF34_1384
ECF34_3302
ECF35_1119
ECF35_3582
ECF36_1595
ECF36_3196
ECF37_2513
ECF37_3390
ECF38_1322
ECF38_1442
ECF39_1438
ECF39_2973
ECF40_1380
ECF40_3198
ECF41_491
ECF41_1141
ECF42_3583
ECF42_4454
ECF43_3477
ECF43_4437
OD (% of WT)
100
75
50
0
0
10
100
IPTG (μM)

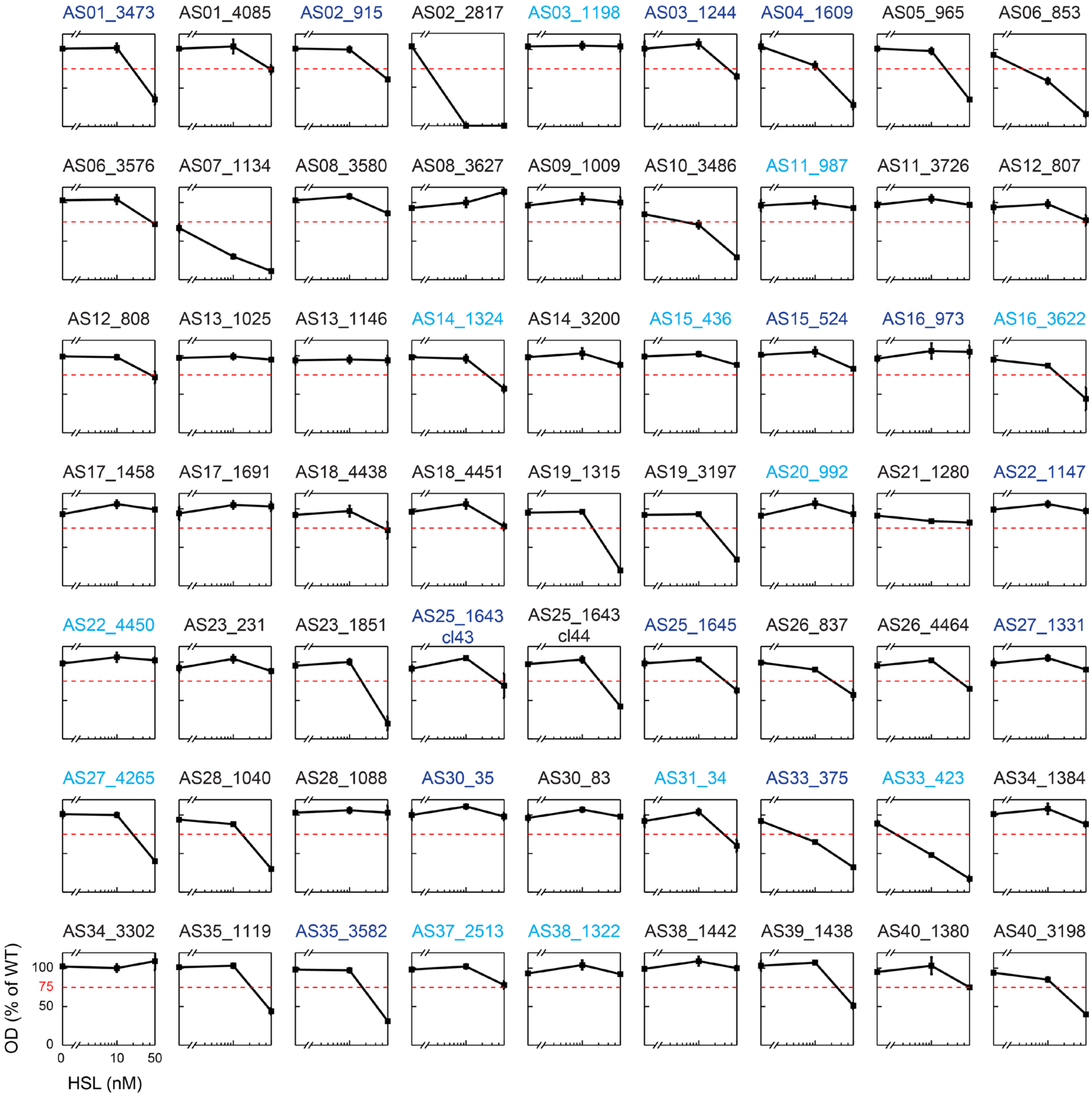

***<u>Figure S9:</u>* Anti-σ transition phase growth assay.** *OD_{600} measurements were taken after 8 hours of growth at different levels of expression of each of the anti-σs in the library. Plots represents the average measurement from three independent assays normalized to cells not expressing an anti-σ, and error bars represent one standard deviation. Dark blue titles indicate the 25 anti-σs tested for orthogonality (Fig. S7), and light blue titles indicate anti-σs that repress σs in the final orthogonal set (Figure 5c).*

II.F. Simultaneous expression of multiple σs

Two σs from the library were simultaneously expressed to test whether they would interfere with each other's function. One relatively high activity σ (ECF20_992) and one relatively low activity σ (ECF34_1384) were selected, and a plasmid constructed that expresses each from a pT7 promoter (pTSc04). This plasmid was transformed into Z-competent *E. coli* DH10β cells (see chimeric σ assay for method) containing plasmid pN565 and either the pECF20_992 or pECF18_up1700 (activated by ECF34_1384) reporters (pVRb plasmids) and induced fully (diluted 1:200 into LB + Antibiotics + 100 µM IPTG, grown at 1000 rpm, 37 °C). The activity and toxicity of the two σs was compared to cells with a negative control plasmid (pET21a) and plasmids expressing only one σ (pVRa plasmids).

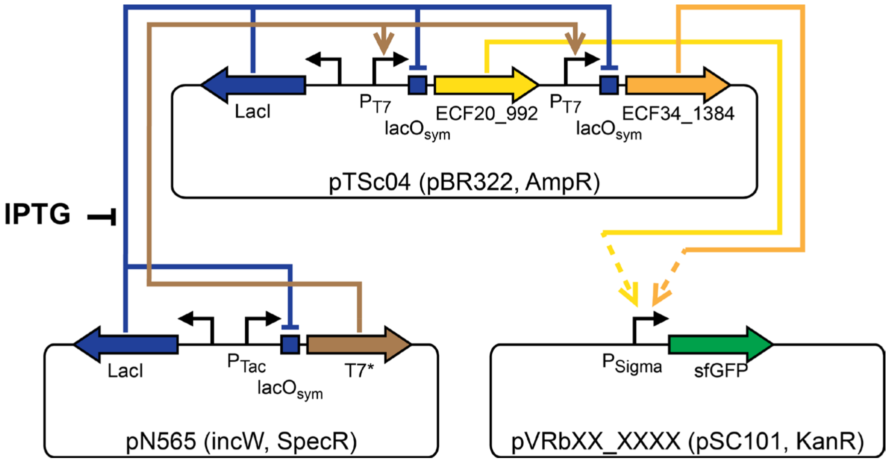

Figure S10: ***Plasmids for simultaneous expression of multiple σs.*** *pTSc04 is based on pVRa, with both ECF20_992 and ECF34_1384 expressed from pT7 promoters. It is driven by T7 RNA polymerase from pN565, and the activity of the σs measured using plasmid series pVRb.*

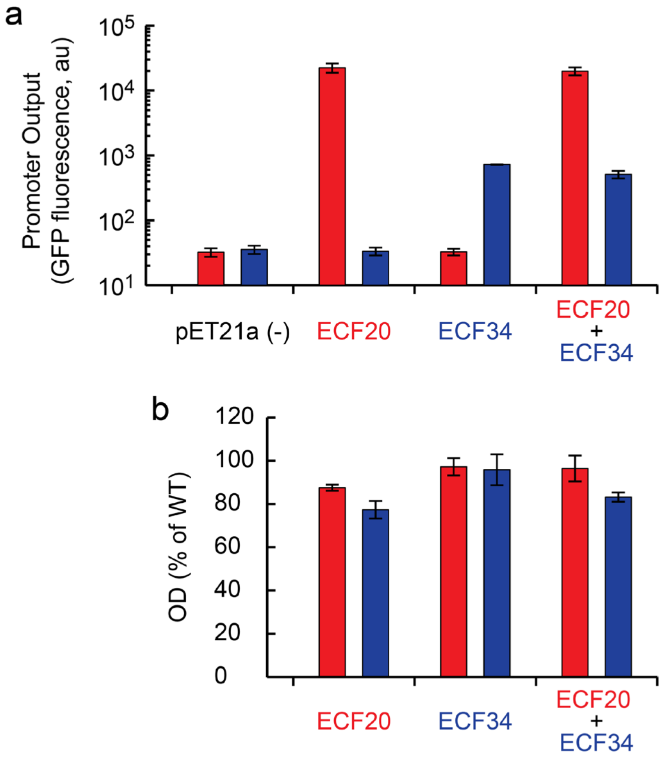

Figure S11: ***Simultaneous expression of multiple σs.*** *Cells carrying either the reporter for ECF20_992 (p20_992, red bars) or for ECF34_1384 (p18_up1700, blue bars) were transformed and assayed with plasmids expressing either ECF20_992, ECF34_138, both of them simultaneously, or a negative control.* ***(a)*** *After induction with 100 µM IPTG for 6 hours, fluorescence was measured with flow cytometry to gauge σ activity.* ***(b)*** *After a total of 8 hours of induction, OD_{600} measurements were taken with a Synergy H1 plate reader and normalized to cells not expressing a σ. For both measurements, each bar represents the mean from three independent experiments, and error bars show one standard deviation.*

II.G.   Expression of a σ in Klebsiella oxytoca

A strongly active σ, ECF20_992, was expressed in *Klebsiella oxytoca* M5a1 to assess how the σs may perform in a different species. The σ library plasmid expressing ECF20_992 (pVRa20_992) was transformed into Z-competent *Klebsiella oxytoca* M5a1 cells (see chimeric σ assay for method) containing pN565 and the ECF20_992 reporter (pVRb20_992), induced to a moderate amount (diluted 1:200 from overnights into LB + Antibiotics + 10 µM IPTG, grown at 1000 rpm, 30 °C), and the fluorescence and OD600 measured and compared to cells transformed with a negative control plasmid (pET21a). Simultaneous measurements were done with *E.coli* DH10β in an identical fashion (except grown at 37 °C) for comparison.

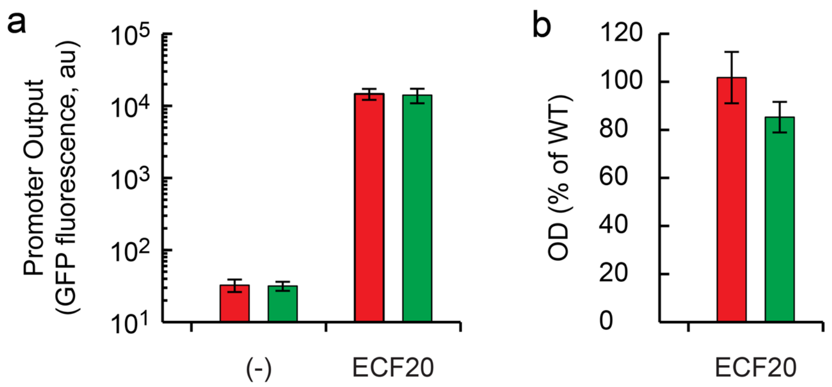

Figure S12: ***Expression of an σ in Klebsiella oxytoca M5a1.*** *The activity and toxicity of ECF20_992 was assayed in both E.coli* DH10β (red bars) *and K.oxytoca* M5a1 (green bars) *carrying a p20_992 reporter plasmid.* ***(a)*** *After 6 hours of induction, fluorescence was measured with flow cytometry.* ***(b)*** *OD_{600} was measured after 8 hours of induction with a Synergy H1 plate reader and normalized to cells carrying a negative control plasmid. All bars represent the mean from three independent experiments and error bars show one standard deviation.*

II.H. Characterization of induction plasmids

In order to characterize the relative strengths of the expression systems in our assays, plasmids were constructed that express sfgfp in place of a σ or anti-σ. (Figure S13). These plasmids were induced using IPTG (0, 4, 10, 20, and 100 μM) or HSL (0, 1, 5, 10, and 50 nM) as appropriate and the fluorescence resulting from GFP expression was measured.

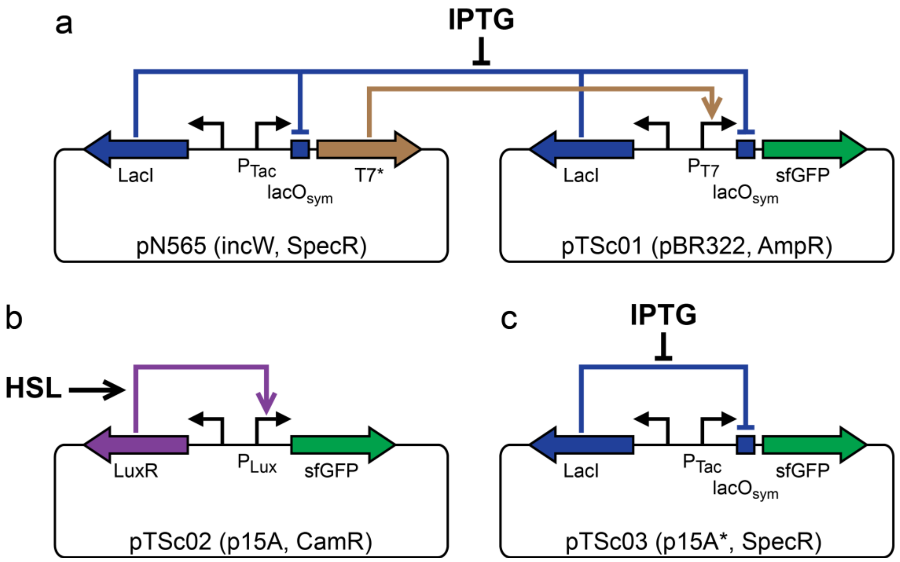

Figure S13: **Plasmids used for expression system characterization. *(a)*** *pTSc01 is based on the pVRa plasmid series with sfGFP expressed in place of a σ.* ***(b)*** *pTSc02 is based on the pVRc plasmid series with sfGFP expressed in place of an anti-σ.* ***(c)*** *Plasmid pTSc03 is based on the pTSa plasmid series with sfGFP expressed in place of a parental or chimeric σ.*

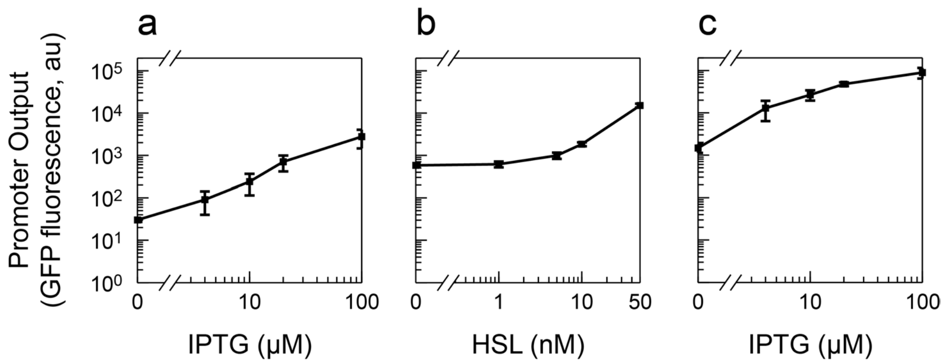

Figure S14: **Induction plasmid transfer functions. *(a)*** *E.coli DH10β cells carrying pTSc01 and Nif565 were induced with IPTG (0, 4, 10, 20, and 100 μM) for 6 hours.* ***(b)*** *E.coli DH10β cells carrying pTSc02 were induced with HSL (0, 1, 5, 10, and 50 nM) for 6 hours.* ***(c)*** *E.coli DH10β cells carrying pTSc03 were induced with IPTG (0, 4, 10, 20, and 100 μM) for 6 hours. In all cases, fluorescence was measured with flow cytometry, and data points represent the average of three measurements taken on separate days, with error bars showing one standard deviation.*

III. Quantification of Anti-σ Threshold Control

A subset of the anti-σ:σ pairs were assayed in more detail to determine their capability to implement ultrasensitivity through sequestration[17] (Figure S15). Sixteen of the σ:anti-σ:promoter sets previously tested were selected based on either: 1. targeting one of the σs in the orthogonal subset, or 2. having an induction curve that suggests switch-like behavior in Figure S6. These sets were induced at four levels (0, 5, 25, and 100 µM IPTG) of σ and three levels (no anti-σ plasmid, 0 nM HSL induction, 50 nM HSL induction) of anti-σ and the promoter activities were measured via fluorescence. High expression of the anti-σ often significantly reduced the promoter output at all levels of σ induction, in many cases also causing highly toxic effects. In contrast, the lower anti-σ induction showed the desired threshold effect in many cases. At this level of anti-σ, the higher levels of σ induction showed promoter activity close to the no anti-σ control, while the lower levels of σ had much lower activity than the equivalent induction points with no anti-σ. This differential repression is characteristic of a threshold system, and increases the utility of these proteins in applications where a more digital-like signal response is desired. The 9 anti-σ:σ:promoter sets that have the best induction curves and correspond to an orthogonal σ are shown in Figure 5d.

Figure S15 (next page): ***Anti-σ thresholding.*** *Sixteen sets of anti-σ, σ, and promoter were assayed at varying inductions of both anti-σ and σ to characterize their ability to implement thresholding. The anti-σs were either not present (blue), or induced at 0nM HSL (green) or 50nM HSL (red), while the σs were induced at 0, 5, 25, and 100 µM IPTG. Plots represent the average promoter activity from three independent assays and error bars represent one standard deviation. Histograms show representative distributions from a 0 and 100 µM induction for each set. Light blue titles indicate data shown in Figure 5d.*

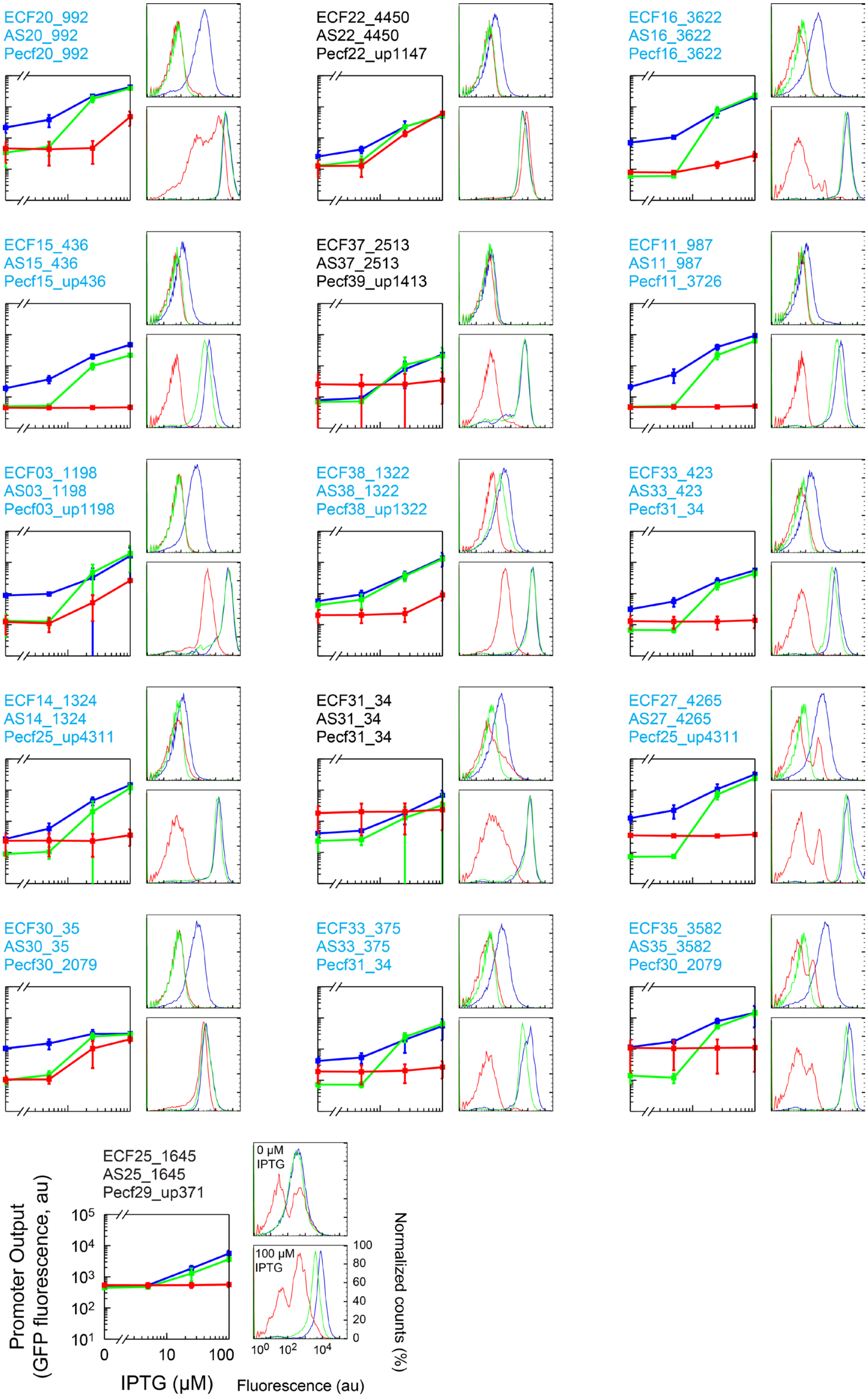
ECF20_992
AS20_992
Pecf20_992
ECF22_4450
AS22_4450
Pecf22_up1147
ECF16_3622
AS16_3622
Pecf16_3622
ECF15_436
AS15_436
Pecf15_up436
ECF37_2513
AS37_2513
Pecf39_up1413
ECF11_987
AS11_987
Pecf11_3726
ECF03_1198
AS03_1198
Pecf03_up1198
ECF38_1322
AS38_1322
Pecf38_up1322
ECF33_423
AS33_423
Pecf31_34
ECF14_1324
AS14_1324
Pecf25_up4311
ECF31_34
AS31_34
Pecf31_34
ECF27_4265
AS27_4265
Pecf25_up4311
ECF30_35
AS30_35
Pecf30_2079
ECF33_375
AS33_375
Pecf31_34
ECF35_3582
AS35_3582
Pecf30_2079
ECF25_1645
AS25_1645
Pecf29_up371
Promoter Output
(GFP fluorescence, au)
10^5
10^4
10^3
10^2
10^1
0
10
100
IPTG (μM)
0 μM
IPTG
100 μM
IPTG
100
80
60
40
20
0
10^0
10^2
10^4
Fluorescence (au)
Normalized counts (%)

To precisely model the impact of the anti-σ sequestration on switch cooperativity, a threshold-gated switch was constructed using ECF20_992 and AS20_992 and characterized more thoroughly (Figure S17). The inducible anti-σ system was supplemented by a set of plasmids constitutively expressing the anti-σ AS20_992 at a number of levels (Figure S16). Changing the strength of a constitutive promoter allowed for finer control over the expression level of anti-σ. This system was tested at 8 induction levels of the σ (0, 5, 25, 50, 75, 100, 150, 200 µM IPTG) to characterize the transfer function. Finally, the Hill equation was used to fit the data,

$$\frac{y-y_{min}}{y_{max}-y_{min}} = \frac{x^n}{x^n+K^n} \quad , \qquad \text{(S5)}$$

where x is the IPTG induction concentration, y is the output (promoter activity), y_{max} is the maximum output, y_{max} is the minimum output, K is the half-maximum, and n is the Hill coefficient. The optimization was weighted to minimize the relative least-squares error so that the model fit both the low and high ends of the data.

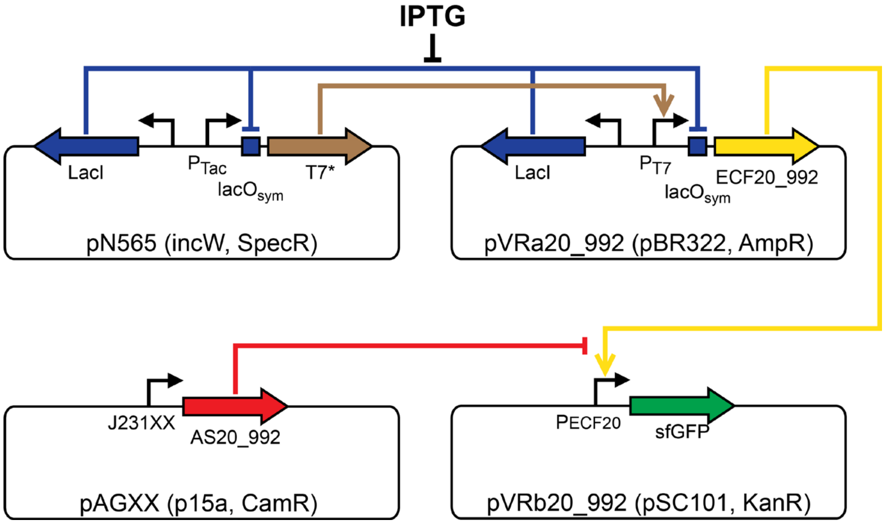

Figure S16: ***Alternate plasmid set used in anti-σ threshold experiments.*** *An alternate series of plasmids, pAGXX, replaces the pVRcXX_XXXX plasmid series used the anti-σ library testing experiments (Fig. S23). This plasmid series expresses the anti-σ AS20_992 from a range of constitutive promoters instead of from the P_{lux} promoter.*

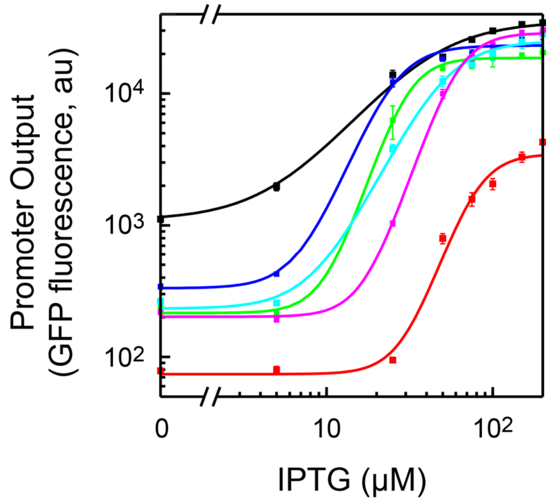

Figure S17: ***Hill fits to AS/ECF20_992 anti-σ:σ threshold switch.*** *Threshold-gated switches were built using σ ECF20_992 and its corresponding anti-σ and promoter. ECF20_992 was induced with varying levels of IPTG (0, 5, 25, 50, 75, 100, 150, and 200 µM) in the presence of plasmids expressing AS20_992 from varying promoters: No anti-σ (black), BBa_J23117 (blue), BBa_J23105 (green), BBa_J23101 (cyan), BBa_J23100 (pink), pLux induced with 50 nM HSL (red). Equation S5 was fit to the data using a relative least-squares algorithm and the fit is shown. The Hill coefficient n is higher when the anti-σ is present: n = 1.7 (No anti-σ), n = 3.3 (BBa_J23117), n = 4.1 (BBa_J23105), n = 2.6 (BBa_J23101), n = 3.9 (BBa_J23100 and pLux). Plots represent the average promoter activity from three technical replicates (No anti-σ) or three independent assays (all others) and error bars represent one standard deviation.*

IV. Creating Chimeric σs

IV.A. Design of chimeric σs and promoters

A combination of protein alignment, structural information, and secondary structure prediction algorithms were used to generate chimeric σs from ECF02_2817 and ECF11_3726 (Figures 3g and S18a). These parental σs were chosen since they have high activity in *E. coli* and there is protein structural information is available that could be used to guide the construction of the chimeras (ECF02_2817 (*E. coli* σ^E)[18] and *R. sphaeroides* σ^E, which belongs to the same subgroup as ECF11_3726[19]). Chimeras of both combinations (N-terminal ECF02_2817 / C-terminal ECF11_3726 and N-terminal ECF11_3726 / C-terminal ECF02_2817) were created by recombining the parental proteins at six 'crossover seams' located in the flexible linker region between the conserved domains 2 and 4, which recognize the -10 and -35 promoter subsites, respectively. While domains 2 and 4 play the most important roles in promoter recognition, the linker region between these regions in Group I σs plays an important role in abortive initiation and promoter escape[20], and likely plays a similar role in the ECF σs. Consequently, the choice of crossover seams within the linkers of the ECF02_2817 and ECF11_3726 σs may affect the functionality of the resultant chimeras. The structure and precise boundary of the linker region in the ECFσs is ambiguous for two reasons: 1. in both structures the σs are bound to their cognate anti-σ, distorting the structure of the linker and 2. the amino acid sequence of the linker region is poorly conserved, making accurate alignments challenging.

In order to select a range of potentially functional crossover seams, the full library of 86 σs was initially aligned using clustalW (http://www.ebi.ac.uk/Tools/msa/clustalw2/)[21]. The alignment of ECF02_2817 and ECF11_3726 was then tweaked by hand based on the protein structures mentioned previously. Crossover seams 1 and 2 were located at either end of the flexible linker in this alignment. Due to some uncertainties in the structural analysis (specifically, that the linkers were too distorted by binding anti-σs for proper structural analysis) crossover seams 4, 5, and 6 were generated from the unaltered clustalW alignment near the beginning, middle, and end of the linker. Finally, a secondary structure prediction algorithm, PredictProtein[22], was used to analyze ECF02_2817 and ECF11_3726 for α-helices. Crossover seam 6 was placed one residue before the beginning of the first α-helix after the linker region in both proteins.

Chimeric promoters were similarly created by crossing over cognate promoters for ECF02_2817 and ECF11_3726 between the -10 and -35 boxes (Figure S18b). The promoter rpoHP3[23] from *E. coli* was used as the parental pECF02 promoter, with a 1 bp mutation (T-34G) made from the WT sequence to differentiate it more from ECF11 promoters. (Note that this promoter contains an overlapping σ70 promoter[23], which likely partly accounts for the high background induction level and low dynamic range of activation by ECF02.) The pECF11_3726 promoter from the σ library was chosen as the parental pECF11 promoter. In each case, to -60 to +20 region of the promoter was used, and these parental promoters were crossed over between -20 and -21 to make chimeric promoters. While the initially engineered chimeric promoters were functional, they were relatively weak when compared to the parental promoters. One explanation for reduced activity is that while the -10 and -35 recognition sites are identical to the parental plasmids, the spacing between them may not be optimal for the chimeric proteins. This is made even more likely because of uncertainties in identifying the -10 and -35 sites in the promoter, and because the ECF02 and 11 promoter models have different optimal spacings (Figures 2 and S1: ECF02 has optimal spacing 14, and ECF11 has optimal spacing 16). For these reasons, additional chimeric promoters were engineered with the -10 and -35 sites moved either 1bp closer or 1bp farther apart.

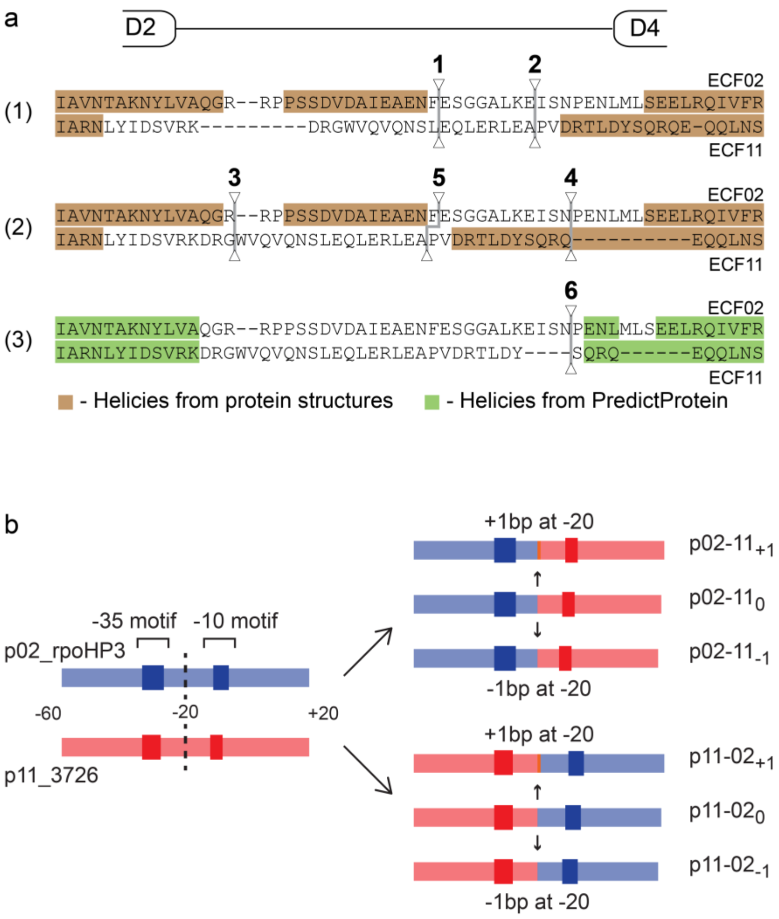

Figure S18: **_Chimeric σ and promoter engineering. (a)_** *Three alignments of the flexible linker region connecting domains 2 and 4 of the parental σs are shown with the crossover seams derived from each alignment marked. Alignment 1 was generated through protein sequence alignment, hand-adjusted based on crystal structures. Alignment 2 was generated through protein sequence alignment alone. Alignment 3 was created with secondary structure prediction by PredictProtein. Each of the six marked seams was used to engineer one chimeric σ of either orientation, for a total of 12 chimeric σs.* ***(b)*** *Three chimeric promoters of each orientation were similarly engineered. pECF02_rpoHP3 and pECF11_3726 were recombined between -20 and -21 to make p02-11$_0$ and p11-02$_0$. To correct for any differences in optimal spacing between the chimeras and parental σs, 1 bp was added or removed at the -20/-21 seam to make additional promoters with longer or shorter spacers.*

IV.B. Chimeric σ characterization

The chimeric σs and promoters were first assayed to determine which crossover seams and promoter variants were most successful (Figure S19). Each of the six versions of each chimera was paired with each of the three versions of its cognate promoter, and the promoter activity determined *in vivo*. From this assay, it seems as though σs can tolerate chimeragenesis in many different areas and alignments within the linker. Despite the differing alignments used to design the chimeras, seams 1-4 produced very active chimeras for both ECF02-11 and ECF11-02, seams 5 and 6 were slightly active in ECF11-02, and seam 6 was slightly active in ECF02-11. Of these seams, seam 1 was chosen for further experimentation as it was the most active variant of ECF11-02, and one of the more active variants of ECF02-11. In contrast to the flexibility on protein crossover location, the chimeric promoter spacing had an extreme effect on σ chimera activity. The initially built chimeric promoters had an intermediate level of activity, while pECF02-11_{-1} and pECF11-02_{+1} were greatly improved. In contrast, pECF11-02_{-1} and pECF02-11_{+1} were inactive, indicating that 1-2 bps of change in the distance between the -10 and -35 sites is enough to abrogate promoter activity. This result also demonstrates that optimal promoter spacing is determined by the source of the parent -10/Domain 2 of the chimeric promoter and σ. Based on these results, ECF11-02 #1, ECF02-11 #1, pECF02-11_{-1} and pECF11-02_{+1} were chosen as the chimeric σs and promoters to be used for further chimera testing.

Next, using the optimized chimera constructs, the parental and chimeric σs and promoters were tested with each other to check their orthogonality (Figures S20, S21, and 3e). Each of the two parental σs and chimeras from the most active seam was tested with each of the two parental promoters and best chimeric promoters. Promoter activity was measured *in vivo*, and fold induction of each promoter was calculated using a negative control plasmid that does not express a σ. This assay demonstrates that both the -10 and -35 sites must be recognized for promoter activation. The chimeric σs activated their promoters by more than 50-fold more than the parental σs, and the parental σ promoters likewise only recognized the parental σs. ECF02_2817:pECF02_rpoHP3 displayed the weakest fold induction at ~4-10-fold, however, this is due to extremely high background activation and toxicity from overexpression. This assay was run in two different strains, *E. coli* CAG22216, which is deficient in ECF02_2817 (a native *E. coli* σ), and *E. coli* DH10β. The results were consistent across both strains and assay conditions, with the chimeric σs remaining orthogonal to each other and their parents.

Finally, the toxicity of the chimeric σs was assayed and compared to the parental σs. The two parental σs and the two chimeras with seam #1 were transformed into *E. coli* DH10β cells carrying a negative control reporter plasmid and the transition phase (8hr) OD measured after induction. The assay shows that neither of the chimeric σs exhibits the extreme toxicity of ECF02_2817, one of their parents.

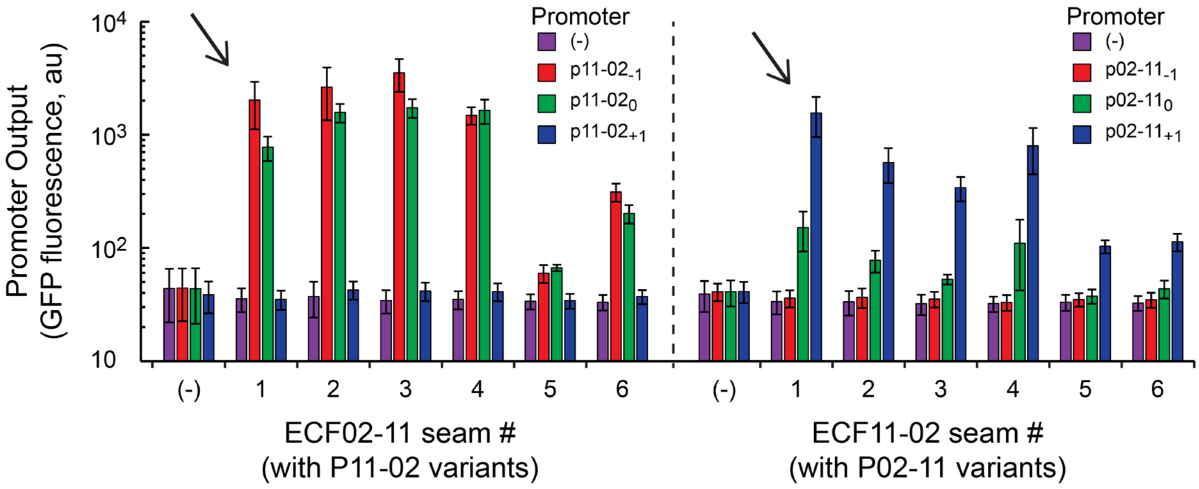

Figure S19: ***Testing of chimeric σ and chimeric promoter variants.*** *Each of the engineered chimeric σs was tested with each of the variants of its cognate chimeric promoter. Negative controls represent an empty plasmid lacking either a σ coding region, or a σ promoter:reporter cassette. Arrows point to the σ chimera: chimera promoter pairs chosen for orthogonality testing. Assays were performed in vivo in E. coli CAG22216 cells at 30°C, induced for 6 hours with 10 μM IPTG, and promoter activity was measured by flow cytometry. Each bar represents the average promoter activity from three independent assays, and error bars represent one standard deviation.*

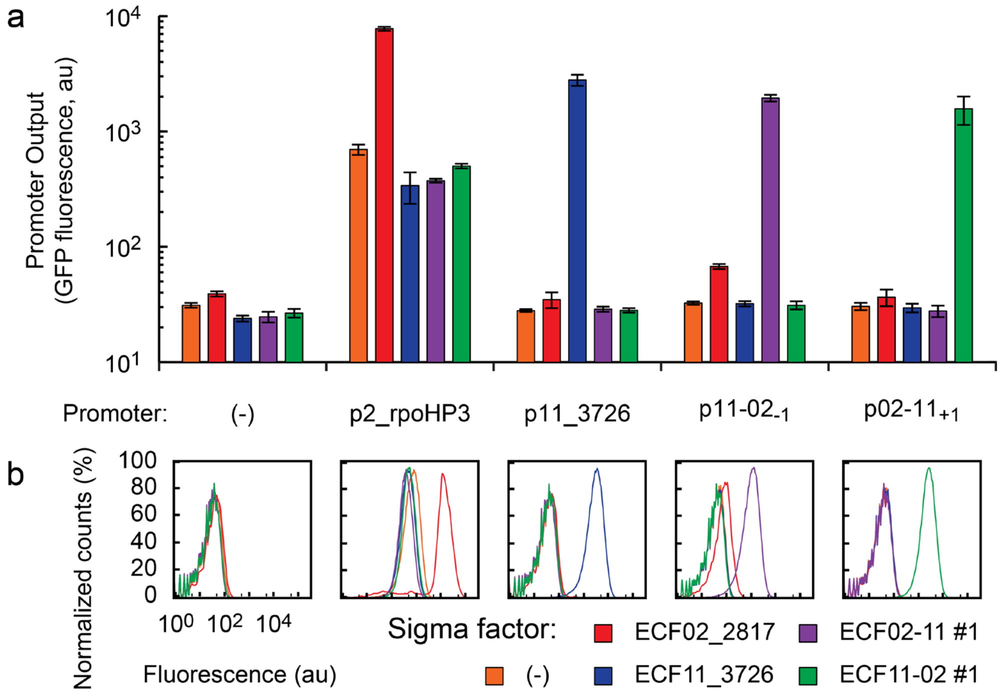

Figure S20: ***Orthogonality testing of chimeric and parental σs in E. coli CAG22216 cells. (a)*** *Initial orthogonality testing of the chimeric σs in E. coli CAG22216 cells and* ***(b)*** *representative cytometry data. Assays were performed in vivo in E. coli CAG22216 cells at 30°C, induced for 6 hours with 10 μM IPTG, and promoter activity was measured by flow cytometry. (-) controls represent cells carrying a plasmid identical to the chimeric σ expression or reporter plasmids, but with the chimeric σ gene, or promoter/reporter removed. Each bar represents the average promoter activity from three three independent assays, and error bars represent one standard deviation. Histograms show representative flow cytometry distributions for each promoter in the presence of all of the σs.*

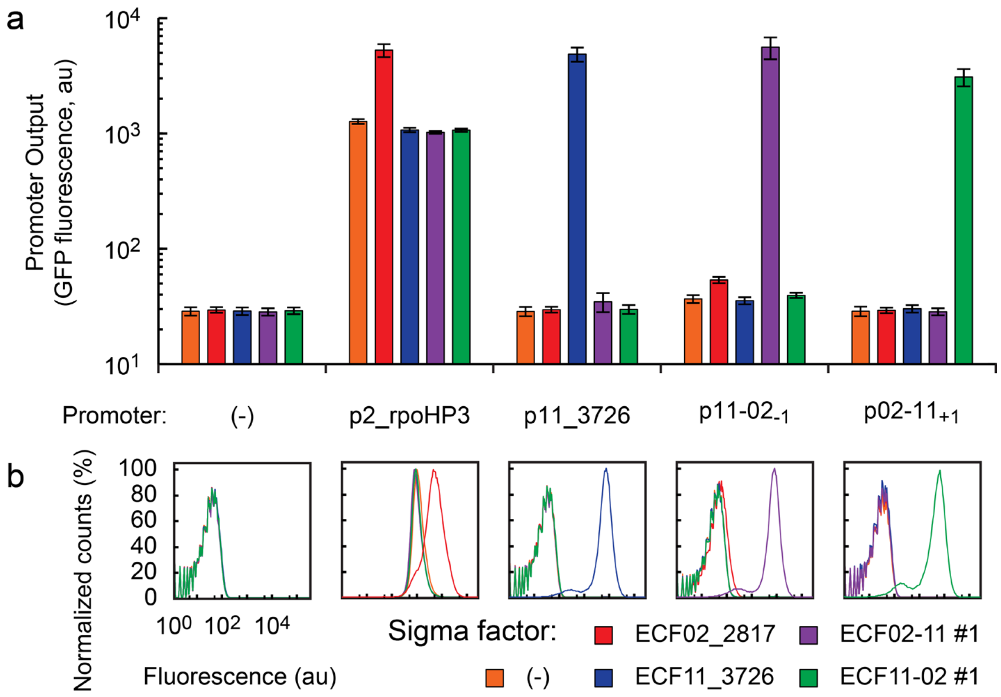

<u>Figure S21:</u> **_Orthogonality testing of chimeric and parental σs in E. coli DH10β cells._** **(a)** *Promoter output data corresponding to Figure 3G.Each bar represents the mean fluorescence from three independent assays measured after 6 hours of induction with 10 µM IPTG. Error bars show the standard deviation.* ***(b)*** *Representative cytometry distributions from the assay showing each promoter in the presence of all of the σs.*

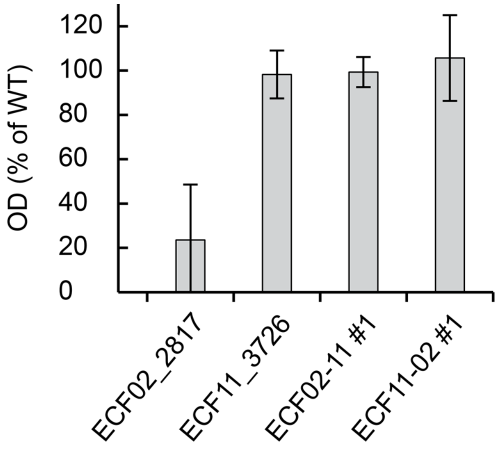

<u>Figure S22:</u> ***Toxicity of chimeric σs.*** *Plasmids expressing ECF02_2817, ECF11_3726, the two most active chimeras, and a negative control with no σ were transformed into DH10β cells, induced with 10 µM IPTG, and their OD600 measured after 8 hours. Each bar shows the mean OD from four independent assays normalized to the OD of the cells containing the negative control. Error bars show one standard deviation.*

V. Plasmids

A 4-plasmid system was used for expressing the σ, promoter and anti-σ libraries (Figure S23). Plasmid pN565 encodes an IPTG-inducible low processivity T7 RNA polymerase enzyme. This was used to weakly express the σ library under control of a T7-regulated promoter encoded on the pVRa plasmid series. The pVRb plasmid series carries the σ-dependent promoters fused to the fluorescent reporter, superfolder GFP[24]. The pVRc plasmid series encodes the anti-σ library under control of HSL. Plasmid modifications were performed using Type II restriction enzyme cloning, PCR and one-step isothermal DNA assembly[25]. The σ and anti-σ gene libraries were codon optimized for *E. coli* K12 MG1655, constructed by gene synthesis and assembled into their parent vectors by GeneArt, Life Technologies.

Plasmid pN565 (incW (2-3 copies)[25], SpecR) is a variant of the low processivity T7 RNA polymerase expression vector, pN249[26] and is tightly regulated by IPTG. The plasmid encodes T7 RNAP with a GTG initiation codon for low translation, an N-terminal degradation tag and the active site mutation R632S. T7 RNAP is expressed from a weak RBS sequence tuned to 50 units using the RBS calculator[27] and a modified P_{tac} promoter with a symmetrical LacO operator sequence (aattgtgagcgctcacaatt), enabling near complete promoter repression in the absence of IPTG. The plasmid also encodes LacI.

Plasmid series pVRa (pBR322 (15-20 copies/cell)[28], AmpR) expresses the σ library from a T7-lacO promoter. The plasmids are derived from pET15b (Novagen) in which the thrombin cleavage site was replaced with a PreScission protease cleavage site. The series encodes codon optimized σ genes on *Nde*I-*Hind*III fragments in frame with an N-terminal His_6 tag and intervening PreScission site. The plasmids and amino acid sequences of the σs are listed in Supplementary Table S1.1.

Plasmid series pVRb (SC101 (~5 copies/cell)[28], KanR) carries the σ-dependent promoter library fused to superfolder GFP (*sfgfp*)[24]. The plasmids are derived from the GFP expression vector, pUA66[29], in which the reporter gene *gfpmut2* was replaced with *sfgfp* on a *Bam*HI-*Pst*I fragment. Promoter sequences from -60 to +20 with respect to the transcription start site were inserted upstream of *sfgfp* into the *Bbs*I-*Bam*HI sites of pVRb (the 5' end of the -10 motif was assumed to be at position -10). For each promoter, DNA fragments were assembled from 4 overlapping 45-mer DNA oligos that corresponded to native promoter sequence, and 2 flanking vector specific oligos. The oligos were assembled by PCR to generate 120 bp fragments in which the 80 nt promoter sequence is flanked by 20 nt of vector sequence. The fragments were gel purified and assembled into purified pVRb *Bbs*I-*Bam*HI vector using one-step isothermal DNA assembly. The plasmids and promoter sequences are listed in Supplementary Table S1.2.

Plasmid series pVRc (p15a (10-12 copies/cell)[28], CmR) expresses the anti-σ library from a HSL-regulated P_{lux} promoter. The plasmids contain *cat* and *LuxR* under constitutive control, and replicate via a p15a origin. The plasmids and amino acid sequences of the anti-σs are listed in Supplementary Table S1.3.

A 2-plasmid system was used to test the chimeric σs and their cognate promoters (Figure S24). Plasmid series pTSaXX (p15a*, SpecR) expresses parental (ECF02_2817, ECF11_3726) and chimeric σs under the control of a modified P_{tac} promoter with a symmetrical LacO operator sequence. These plasmids were derived from pSB3C5[30], and contain a mutation in the origin that appears to cause them to be maintained at a higher copy number than wild-type p15a. Plasmid series pTSbXX (pSC101, KanR) contains parental and chimeric σ-dependent promoters driving expression of *sfgfp*. These plasmids are very similar to plasmid series pVRb, with only the promoter region varying. All construction of these plasmid series was done with one-step isothermal DNA assembly or PCRs and blunt ligations.

pVRa, pVRb, and pVRc plasmids containing active σs, anti-σs, and promoters will be made available upon request at Addgene (http://www.addgene.org/Christopher_Voigt/).

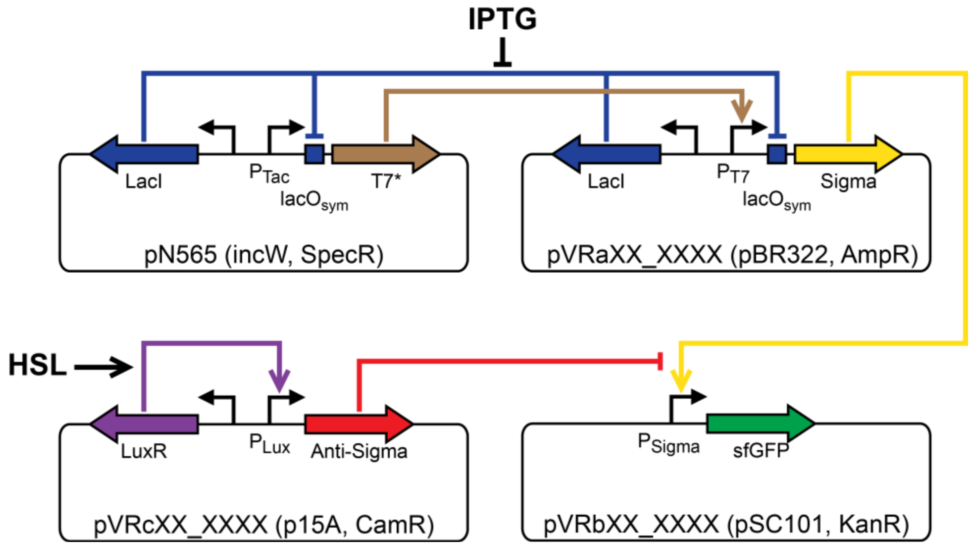

***<u>Figure S23:</u>* Plasmids used for σ and anti-σ characterization.** *Low processivity T7 RNA polymerase (T7*) is expressed from pN565 using an IPTG-inducible P_{tac} promoter with a symmetric lac operator (lacO_{sym}). T7* is used to express the σ library via a T7 and IPTG induced promoter (consisting of the P_{T7} promoter sequence followed by a symmetric lac operator) from the pVRaXX_XXX plasmid series. σ-dependent promoters ($P_σ$) are carried on the pVRbXX_XXX plasmid series fused to superfolder gfp. The anti-σ library carried on the pVRcXX_XXX plasmid series is under HSL control via the P_{lux} promoter. XX_XXX in each of the library names represents which anti-σ / σ / or σ-dependent promoter the plasmid carries. For example, the set pVRa20_992, pVRb20_992, pVRc20_992 carries ECF20_992, AS20_992, and pECF20_992, respectively.*

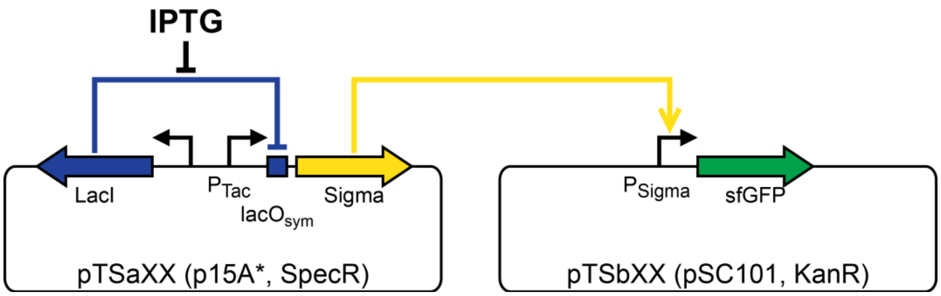

***<u>Figure S24:</u>* Plasmids used for chimeric σ characterization.** *A two plasmid system was used to characterize the chimeric σs. Plasmid library pTSaXX expresses the parental or chimeric σs under the control of an IPTG inducible P_{Tac} promoter with a symmetric lac operator (lacO_{sym}). Plasmid library pTSbXX contains the parental and chimeric σ-dependent promoters driving sfgfp.*

VI. Supplementary Tables

The following tables are included as separate files:

Supplementary Table 1 – Libraries and plasmids.

1.1 – σ library. A summary of the full σ library, including source organism, sequence, and plasmid name.
1.2 – Promoter library. Details of the promoter library, including sequences and plasmid names.
1.3 – Anti-σ library. A summary of the anti-σ library, including source organism, sequence, and plasmid name.
1.4 – Plasmids. A list of all plasmids used.

Supplementary Table 2 – σ and anti-σ library data.

2.1 – σ orthgonality. The full map of σ : promoter interactions that is shown in Figure 3e and Supplementary Figure S4. Includes average fold induction values, standard deviations, and the number of replicates for each point.

2.2 – σ titrations. Induction curves of 52 active σs from the library against their most active target promoter. This data is shown in Supplementary Figure S5.

2.3 – Anti-σ orthogonality. The full map of anti-σ : σ interactions that is shown in Figure 5c and Supplementary Figure S7. Includes average fold induction values, standard deviations, and the number of replicates for each point.

2.4 – σ growth assays. Three measures of the toxicity of the σ library: exponential growth (Supplemental Figure S8), transition phase OD, and colony size on solid media.

2.5 – Anti-σ growth assays. Three measures of the toxicity of the anti-σ library: exponential growth (Supplemental Figure S9), transition phase OD, and colony size on solid media.

Supplementary Table 3 – Analysis of natural σ occurrences.

3.1 – Analysis of co-occurrence. The data from Tables 3.2 and 3.3 is used to statistically determine whether orthogonal σ subgroups are more likely to naturally be found with other σ subgroups.

3.2 – ECF σ co-occurrence. Data from Staron, et. al.[1] was used to generate a table of how frequently the different subgroups of ECF σs occur in the same genome.

3.3 – Natural sigma orthogonality. The data from Table 3.2 and our σ orthogonality map was used to determine which subgroups of σs do not crosstalk with the other subgroups they are found with.

3.4 – Examples of natural σ cascades. Our crosstalk analysis and 3.2 is used to predict a few examples of subgroups of σs that may form cascades.

VII. References

1. Staroń, A. *et al.* The third pillar of bacterial signal transduction: classification of the extracytoplasmic function (ECF) σ factor protein family. *Mol. Microbiol.* **74,** 557–581 (2009).
2. Rhodius, V. A., Suh, W. C., Nonaka, G., West, J. & Gross, C. A. Conserved and Variable Functions of the σE Stress Response in Related Genomes. *PLoS Biol* **4,** e2 (2005).
3. Liu, X., Brutlag, D. L. & Liu, J. S. BioProspector: discovering conserved DNA motifs in upstream regulatory regions of co-expressed genes. *Pac. Symp. Biocomput. Pac. Symp. Biocomput.* 127–138 (2001).
4. Rhodius, V. A. & Mutalik, V. K. Predicting strength and function for promoters of the Escherichia coli alternative sigma factor, σE. *Proc. Natl. Acad. Sci.* **107,** 2854–2859 (2010).
5. Staden, R. Computer methods to locate signals in nucleic acid sequences. *Nucleic Acids Res.* **12,** 505–519 (1984).
6. Crooks, G. E., Hon, G., Chandonia, J.-M. & Brenner, S. E. WebLogo: A Sequence Logo Generator. *Genome Res.* **14,** 1188–1190 (2004).
7. Salgado, H. *et al.* RegulonDB v8.0: omics data sets, evolutionary conservation, regulatory phrases, cross-validated gold standards and more. *Nucleic Acids Res.* **41,** D203–D213 (2013).
8. Shultzaberger, R. K., Chen, Z., Lewis, K. A. & Schneider, T. D. Anatomy of Escherichia coli σ70 promoters. *Nucleic Acids Res.* **35,** 771–788 (2007).
9. Yuzenkova, Y., Tadigotla, V. R., Severinov, K. & Zenkin, N. A new basal promoter element recognized by RNA polymerase core enzyme. *EMBO J.* **30,** 3766–3775 (2011).
10. Burr, T., Mitchell, J., Kolb, A., Minchin, S. & Busby, S. DNA sequence elements located immediately upstream of the –10 hexamer in Escherichia coli promoters: a systematic study. *Nucleic Acids Res.* **28,** 1864–1870 (2000).
11. Mitchell, J. E., Zheng, D., Busby, S. J. W. & Minchin, S. D. Identification and analysis of 'extended –10' promoters in Escherichia coli. *Nucleic Acids Res.* **31,** 4689–4695 (2003).
12. Rhodius, V. A., Mutalik, V. K. & Gross, C. A. Predicting the strength of UP-elements and full-length E. coli σE promoters. *Nucleic Acids Res.* **40,** 2907–2924 (2012).
13. Chang, A. C. & Cohen, S. N. Construction and characterization of amplifiable multicopy DNA cloning vehicles derived from the P15A cryptic miniplasmid. *J. Bacteriol.* **134,** 1141–1156 (1978).
14. Nitta, T., Nagamitsu, H., Murata, M., Izu, H. & Yamada, M. Function of the ςE Regulon in Dead-Cell Lysis in Stationary-Phase Escherichia coli. *J. Bacteriol.* **182,** 5231–5237 (2000).
15. Hiratsu, K., Amemura, M., Nashimoto, H., Shinagawa, H. & Makino, K. The rpoE gene of Escherichia coli, which encodes sigma E, is essential for bacterial growth at high temperature. *J. Bacteriol.* **177,** 2918–2922 (1995).
16. Peñas, A. D. L., Connolly, L. & Gross, C. A. SigmaE is an essential sigma factor in Escherichia coli. *J. Bacteriol.* **179,** 6862–6864 (1997).
17. Buchler, N. E. & Cross, F. R. Protein sequestration generates a flexible ultrasensitive response in a genetic network. *Mol. Syst. Biol.* **5,** (2009).
18. Campbell, E. A. *et al.* Crystal Structure of Escherichia coli σE with the Cytoplasmic Domain of Its Anti-σ RseA. *Mol. Cell* **11,** 1067–1078 (2003).
19. Campbell, E. A. *et al.* A Conserved Structural Module Regulates Transcriptional Responses to Diverse Stress Signals in Bacteria. *Mol. Cell* **27,** 793–805 (2007).
20. Murakami, K. S., Masuda, S. & Darst, S. A. Structural Basis of Transcription Initiation: RNA Polymerase Holoenzyme at 4 Å Resolution. *Science* **296,** 1280–1284 (2002).
21. Larkin, M. A. *et al.* Clustal W and Clustal X version 2.0. *Bioinformatics* **23,** 2947–2948 (2007).
22. Rost, B., Yachdav, G. & Liu, J. The PredictProtein server. *Nucleic Acids Res.* **32,** W321–W326 (2004).
23. Erickson, J. W., Vaughn, V., Walter, W. A., Neidhardt, F. C. & Gross, C. A. Regulation of the promoters and transcripts of rpoH, the Escherichia coli heat shock regulatory gene. *Genes Dev.* **1,** 419–432 (1987).

24. Pédelacq, J.-D., Cabantous, S., Tran, T., Terwilliger, T. C. & Waldo, G. S. Engineering and characterization of a superfolder green fluorescent protein. *Nat. Biotechnol.* **24,** 79–88 (2005).
25. Gibson, D. G. *et al.* Enzymatic assembly of DNA molecules up to several hundred kilobases. *Nat. Methods* **6,** 343–345 (2009).
26. Temme, K., Hill, R., Segall-Shapiro, T. H., Moser, F. & Voigt, C. A. Modular control of multiple pathways using engineered orthogonal T7 polymerases. *Nucleic Acids Res.* (2012). doi:10.1093/nar/gks597
27. Salis, H. M., Mirsky, E. A. & Voigt, C. A. Automated design of synthetic ribosome binding sites to control protein expression. *Nat. Biotechnol.* **27,** 946–950 (2009).
28. Sambrook, J. J. & Russell, D. D. W. *Molecular cloning: a laboratory manual. Vol. 2*. (CSHL Press, 2001).
29. Zaslaver, A. *et al.* Just-in-time transcription program in metabolic pathways. *Nat. Genet.* **36,** 486–491 (2004).
30. Shetty, R., Endy, D. & Knight, T. Engineering BioBrick vectors from BioBrick parts. *J. Biol. Eng.* **2,** 5 (2008).
